# P38 inhibition reverses TGFβ1 and TNFα-induced contraction in a model of proliferative vitreoretinopathy

**DOI:** 10.1038/s42003-019-0406-6

**Published:** 2019-05-03

**Authors:** Lauren Schiff, Nathan C. Boles, Marie Fernandes, Bar Nachmani, Ronald Gentile, Timothy A. Blenkinsop

**Affiliations:** 1 Icahn School of Medicine at Mount Sinai, New York, NY 10029 USA; 2 Black Family Stem Cell Institute, New York, NY 10029 USA; 3 Neural Stem Cell Institute, Rensselaer, NY 12144 USA

**Keywords:** Mechanisms of disease, Collective cell migration

## Abstract

Proliferative vitreoretinopathy (PVR) is a metaplasia in the vitreous of the eye manifested by the transformation of retinal pigment epithelial (RPE) cells and the development of contracting epiretinal membranes (ERM), which lead to retinal detachment and vision loss. While TGFβ1 and TNFα have been associated with PVR, here we show that these cytokines act synergistically to induce an aggressive contraction phenotype on adult human (ah)RPE. Connected RPE detach upon contraction and form motile membranes that recruit more cells. TGFβ1 and TNFα (TNT)-induced contracting membranes uniquely express muscle and extracellular rearrangement genes. Whole transcriptome RNA sequencing of patient-dissected PVR membranes showed activation of the p38-MAPK signaling pathway. Inhibition of p38 during TNT treatment blocks ahRPE transformation and membrane contraction. Furthermore, TNT-induced membrane contractility can be reversed by p38 inhibition after induction. Therefore, targeting the p38-MAPK pathway may have therapeutic benefits for patients with PVR even after the onset of contracting ERMs.

## Abstract (web summary)

Schiff et al. show that coactivation of TGFβ1 and TNFα causes aggressive contraction of human retinal pigment epithelial cells and increases expression of genes coding for muscle and extracellular rearrangement proteins. This pathological phenotype is reversed by p38 inhibition, highlighting its potential therapeutic role against proliferative vitreoretinopathy.

## Introduction

Epiretinal membranes (ERMs) are metaplasia near the retina that occur due to activation of quiescent retina cell populations including Muller’s glia, macrophages, and RPE cells^1^. ERMs can be benign and asymptomatic or can be aggressive and exhibit myocontractile behavior, leading to retinal detachments and vision loss^2^. The most aggressive form of ERM formation, Proliferative Vitreoretinopathy (PVR), is thought to derive its severity from activation of RPE, since membranes are composed of up to 95% RPE^3^. Similarly, when RPE are not present, a less aggressive ERM phenotype is observed^4^. We previously discovered a putative multipotent stem cell population in RPE, which may contribute to this observed cellular plasticity^5^.

Development of PVR occurs following a retinal tear and complicates rhegmatogenous retinal detachment, resulting in epiretinal and subretinal membrane formation that can re-detach the retina and lead to blindness. The risk factors for PVR, the primary cause of retinal re-detachment after retinal surgery, include the size of the retinal tear, presence of vitreous hemorrhage, intraocular inflammation and trauma^6–9^. During a retinal tear dislodged RPE come in contact with the vitreous which stimulates RPE migration on the surface of the retina. Inflammatory mediators and blood released from the retinal tear triggers the RPE to become metaplastic and produce collagen, resulting in ERM formation and contraction. Clinically, the retina develops wrinkling and folds which overwhelm the forces keeping the retina attached^10,11^. PVR is also characterized by intraretinal changes caused by retinal gliosis where Müller and other glial cells play an important role^10^.

Although many cytokines, including transforming growth factor beta (TGFβ)^12^ and tumor necrosis factor alpha (TNFα)^13^, have been implicated in retinal tear-induced PVR, what behavioral changes are induced in RPE is not well understood. Both TGFβ and TNFα have been implicated in fibrotic diseases^14–18^. Furthermore, nucleotide polymorphisms in TGFβ1 and TNF loci are known to confer an elevated risk of PVR, suggesting a genetic component^19–21^. TNFα alone has been found to stimulate the production of EMT-associated fibrotic aggregates in ARPE-19, a RPE immortalized cell line^22^ and can affect RPE barrier function via p38 signaling pathway^23^. However, using the same immortalized ARPE-19, another group found both TGFβ1 and TNFα co-stimulation was necessary to produce similar aggregates^24^. The use of ARPE-19 to faithfully predict RPE behavior has come under scrutiny due to reports of not responding like or nor possessing elements of native physiology, leading to reproducibility issues^25–29^.

We sought to characterize the impact of TGFβ and TNFα on a functional ahRPE monolayer with native electrophysiology^30^ and evaluate whether this treatment in vitro evoked changes observed in PVR in vivo. Only upon TGFβ and TNFα co-treatment (TNT), ahRPE form aggregates and their formation is contraction dependent with similar protein composition to patient-dissected PVR membranes, a distinct phenotype not evoked by either TGFβ1 or TNFα treatment alone. Additionally, we identified enrichment of the p38-MAPK pathway in both TNT-treated ahRPE and patient-dissected PVR samples. Lastly, we found inhibition p38-MAPK not only prevents, but can also reverse contractility, potentially the most damaging acquired RPE behavior in PVR.

## Results

### TGFβ1 and TNFα induce ahRPE to form 3D masses

**Fig. 1:**
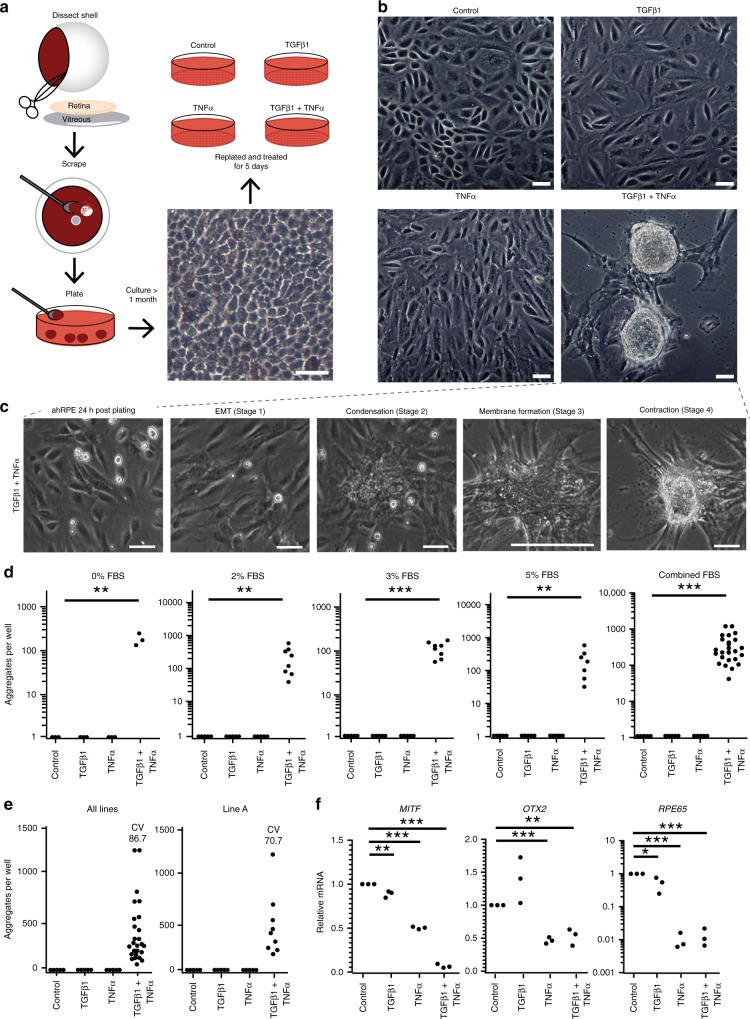
TGFβ1 and TNFα work synergistically to induce transformation of RPE. RPE cultured in the presence of 10 ng/ml TGFβ1 and/or TNFα for 5 days then assayed morphologically and transcriptionally. **a** RPE dissection schematic. Scale bar = 50 μM. **b** Phase images of RPE 5 days after treatment with TGFβ1 and/or TNFα. Scale bar = 50 μM. **c** Time-lapse image frames highlighting stages of RPE transformation when stimulated with TGFβ1 and TNFα for 5 days. Scale bar = 300 μM. **d** Quantification of contractile membranes with varying concentrations of FBS after 5 days treatment with TGFβ1 and/or TNFα. **e** Coefficient of variation (CV) for total RPE lines (n = 11) and one RPE line. **f** RT-qPCR of RPE markers. Statistical significance was calculated using Student’s *t*-test, *P < 0.05; **P < 0.01; and ***P < 0.001

We sought to evaluate the response of ahRPE with native physiology to being dislodged from the epithelial monolayer and exposed to TGFβ1 and TNFα. Following maturation, ahRPE cells were replated and treated with control FBS-basal media, or FBS-basal media supplemented with TGFβ1, or with TNFα, or with the combination of TGFβ1 and TNFα (TNT) (Fig. 1a). Within 5 days, ahRPE treated with TNT-supplemented media underwent a distinct transformation and generated three-dimensional (3D) aggregates (Fig. 1b) resembling those observed in ARPE-19^22,24^. Only in the TNT-treated condition RPE exhibited the formation of aggregates 363.7 ± 61.8, *P* < 0.001, (*n* = 26) per well of a 24 well plate, and neither TGFβ1 0 ± 0, *P* < 0.001, (*n* = 26), nor TNFα alone 0 ± 0, *P* < 0.001, (mean ± S.E.M; *n* = 26) induced a similar ahRPE transformation (Fig. 1d). We repeated the experiment with varying FBS concentration and found aggregate production is independent of FBS since 3D aggregates were formed with 0%, 317.7 ± 57.1, *P* < 0.01, (*n* = 3), 2%, 474.9 ± 138.8, *P* < 0.01, (*n* = 8), 3% 195.5 ± 25.8, *P* < 0.001, (*n* = 8), and 5% FBS 448.6 ± 151.3, *P* < 0.01, (mean ± S.E.M; *n* = 7) (Fig. 1d).

We tested ahRPE lines from 11 different, non-genetically matched donors, and found that 100% of the lines exhibited the formation of aggregates exclusively upon TNT treatment (Fig. 1e). However, in some experiments we only found 42 aggregates, while in others we counted 1200. To determine whether the variability was due to genetic differences between lines or experimental variability, we compared interline variability in membrane number formed upon TNT treatment between all donor ahRPE lines to intraline variability, i.e., variability of the number of membranes formed within individual lines in different experiments. We graphed the interline variability for the line for which we conducted the highest number of experiments (*n* = 6), Line A. A list of the CV for each line is presented in Table S1. The CV across all experiments was 86.7% (n = 26), whereas intraline CV for line A was 70.67% (*n* = 6), suggesting non-genetic influences account for at least 81.5% of the observed membrane formation variability between experiments (Fig. 1e and Supplementary Table 1). By qPCR we also observed reduced expression of the known RPE identity markers, *MITF* 0.069 ± 0.01, *P* ≤ 0.001, (*n* = 3), *OTX2* 0.527 ± 0.07, *P* ≤ 0.01, (*n* = 3), and *RPE65* 0.013 ± 0.01, *P* ≤ 0.001, (mean ± S.E.M; *n* = 3) compared to control (Fig. 1f). Taken together, the data suggest that TNT inhibits RPE identity and drives aggregates formation in RPE independently of genetic background.

### TGFβ and TNFα induced aggregate formation are contraction dependent

**Fig. 2:**
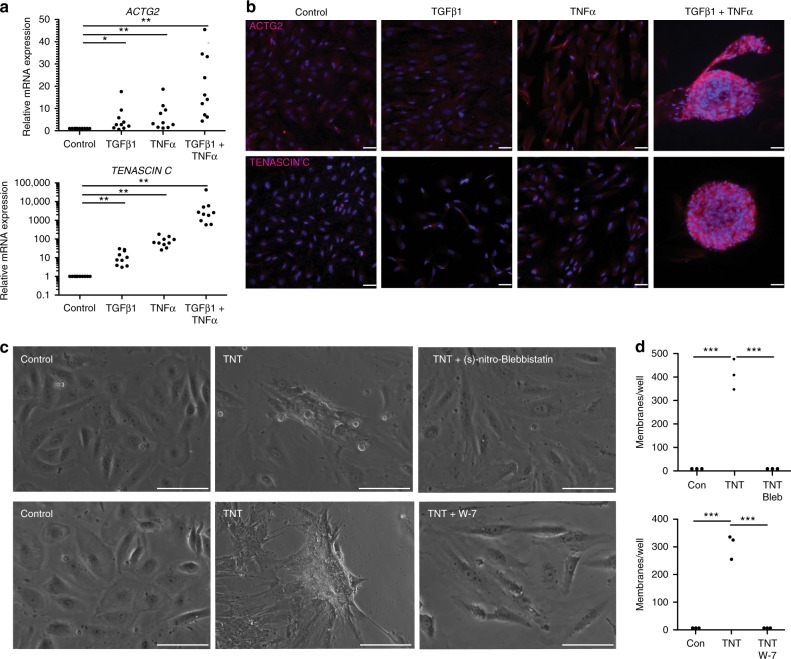
The role of contractility in TGFβ1 and TNFα induced PVR-like membranes. **a** RT-qPCR of extracellular matrix and cell motility markers. **b** Anti-ACTG2 and TENASCIN C immunostaining. Scale bar = 75 μm. **c** Phase images of RPE 5 days after treatment with TNT, TNT + (s)-nitro-Blebbistatin, or TNT + W-7. Scale bar = 50 μm. **d** Quantification of contractile membranes per well after 5 days in control media, TNT media, TNT + (s)-nitro-Blebbistatin media, or TNT + W-7 media. Statistical significance was calculated using Student’s *t*-test, *P < 0.05; **P < 0.01; and ***P < 0.001

Due to the perceived contractile nature of the membranes, we evaluated changes in extracellular matrix reorganization and motility-associated changing genes. The muscle associated actin ACTG2 and the extracellular matrix protein TENASCIN C were both significantly up regulated in TNT-treated ahRPE compared to control, TGFβ1, or TNFα treated RPE (*ACTG2* 19.7 ± 4.4, *P* ≤ 0.01, (*n* = 10) and *TENASCIN C* 6540.6 ± 4063.1, *P* ≤ 0.01, (mean ± S.E.M; *n* = 10) (Fig. 2a) and protein levels (Fig. 2b). We further hypothesized contraction underlies TNT induced aggregation and tested inhibitors of mechanisms involved in contraction. Upon TNT treatment in the presence of (S)-nitro-Blebbistatin, an inhibitor of myosin II ATPases, ahRPE were unable to generate contractile masses (Figs. 2c, d). Additionally, cells treated with TNT in combination with W-7 hydrochloride, a calmodulin antagonist which inhibits myosin light chain kinase, were similarly unable to generate contractile masses (Figs. 2c, d). These results suggest ahRPE aggregation depends on contraction.

To better observe changes between untreated ahRPE, and ahRPE treated with TGFβ1 or TNFα or TNT, we conducted a 72 h-long time-lapse imaging, taking one frame per half hour. In FBS-basal media, ahRPE proliferate and begin to re-establish a monolayer (Supplementary Movie 1) while ahRPE treated with TGFβ1 (Supplementary Movie 2) or TNFα (Supplementary Movie 3) appear more mesenchymal. In TNT supplemented media, ahRPE undergo dramatic morphological and behavioral changes (Supplementary Movie 4). RPE transformation can be divided into four distinct stages (Fig. 1c). First, ahRPE lose high phase edges representative of epithelial cells and begin to appear more mesenchymal (stage 1). Then, cells begin to condense into discrete centers (stage 2). A ridge forms at the edge of these groups suggesting multiple layers we refer to as a membrane (stage 3). Finally, the cluster synchronously contracts to form a contracting membrane (stage 4). These membranes develop more quickly at the edge of the well and contract in discrete, almost rhythmic spurts, eventually ripping cells off the plate (Supplementary Movie 5).

### Differences between TGFβ and TNF cytokine family members

Both TGFβ2 and TNFβ have been implicated in PVR and may also induce a similar response in RPE^12,20^. To determine if cytokine family members behaved similarly, we analyzed ahRPE upon treatment with TGFβ1 + TNFα (TNT), TGFβ2 + TNFα, TGFβ1 + TNFβ and TGFβ2 + TNFβ. Upon 5 days of treatment, all conditions produce robust contracting membranes (Supplementary Fig. 1A) and express PVR associated EMT proteins (Supplementary Fig. 1B). While there was no statistical difference in quantity of contractile membranes produced between treatments, TGFβ1 + TNFα tended to produce the most membranes and express PVR associated EMT genes compared to other combinations (Supplementary Fig. 1C, D). Due to these minimal differences and the implication of TGFβ1 and TNFα in the literature, we continued to explore the effect of TGFβ1 and TNFα.

### The combination of TGFβ1 and TNFα drives ahRPE toward a PVR phenotype

**Fig. 3:**
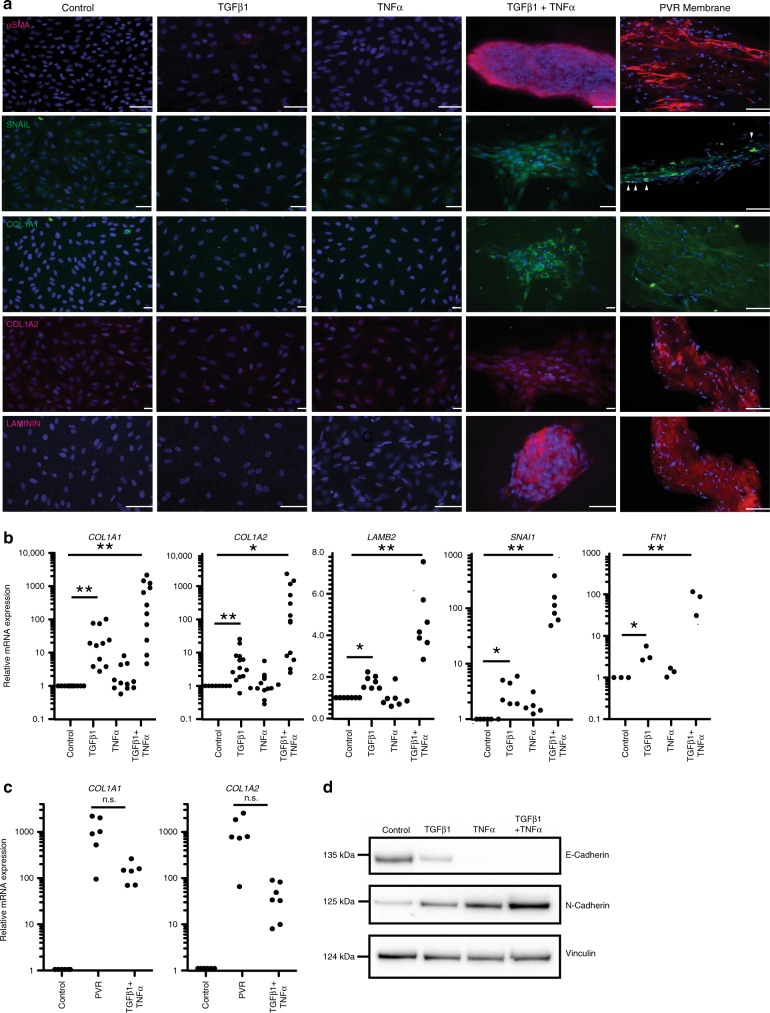
Characterization of TNT-treated ahRPE compared to patient-dissected PVR membranes. **a** Patient-dissected PVR samples and ahRPE were treated with 10 ng/ml TGFβ1 and/or TNFα for 5 days then fixed. Immunostaining was performed with Anti-αSMA (scale bar = 100 μm), SNAIL, COL1A1, COL1A2 (scale bar = 50 μm), and LAMININ (scale bar = 50 μm) antibodies. **b** RT-qPCR of extracellular matrix and EMT specific genes. **c** RT-qPCR of extracellular matrix genes in TNT-treated ahRPE compared to patient-dissected PVR membranes. **d** Western blot analysis comparing E- and N-Cadherin expression in ahRPE treated with TNT-for 5 days. Statistical significance was calculated using Student’s *t*-test, *P < 0.05; **P < 0.01; and ***P < 0.001

Considering the usefulness of an in vitro model of the transformation of RPE during PVR, we sought to compare TNT-treated ahRPE with patient-dissected PVR samples by immunofluorescence imaging and gene expression analysis. We evaluated patient-dissected PVR membranes that an independent clinical pathology lab had confirmed to originate from RPE. By immunofluorescence, all patient-dissected PVR membranes expressed the characteristic EMT markers αSMA and SNAIL as well as the known extracellular matrix proteins^31^ Collagen alpha-2(I) chain (COL1A1), Collagen alpha-2 (II) chain (COL1A2), and LAMININ^32^ similar to RPE treated with TNT (Fig. 3a). TNT treatment also results in increased mRNA expression of *COL1A1* 629.922 ± 219.714, *P* ≤ 0.01, (*n* = 11), *COL1A2* 472.870 ± 204.383, *P* ≤ 0.05, (*n* = 13), *LAMB2* 4.634 ± 0.697, *P* ≤ 0.01, (*n* = 3), *SNAI1* 122.034 ± 40.710, *P* ≤ 0.01, (*n* = 8), and fibronectin, *FN1* 78.294 ± 24.959, *P* ≤ 0.05, (mean ± S.E.M; *n* = 3) compared to untreated and ahRPE treated with either TGFβ1 or TNFα alone (Fig. 3b). Moreover, expression of *COL1A1* and *COL1A2* in patient-dissected PVR membranes was not significantly different compared to TNT-treated ahRPE: *COL1A1* 1128.5 ± 337.7, *P* = 0.0513, (*n* = 6) and *COL1A2* 1615.6 ± 532.0, *P* = 0.0977, (mean ± S.E.M; *n* = 7) (Fig. 3c). Additionally, Western blot analysis demonstrated that upon TNT treatment, there was a shift in expression in ahRPE from E-Cadherin to N-Cadherin, indicating an EMT process^33^ (Fig. 3d).

### p38-MAPK pathway participates in transducing the signal mediated by TNT

**Fig. 4:**
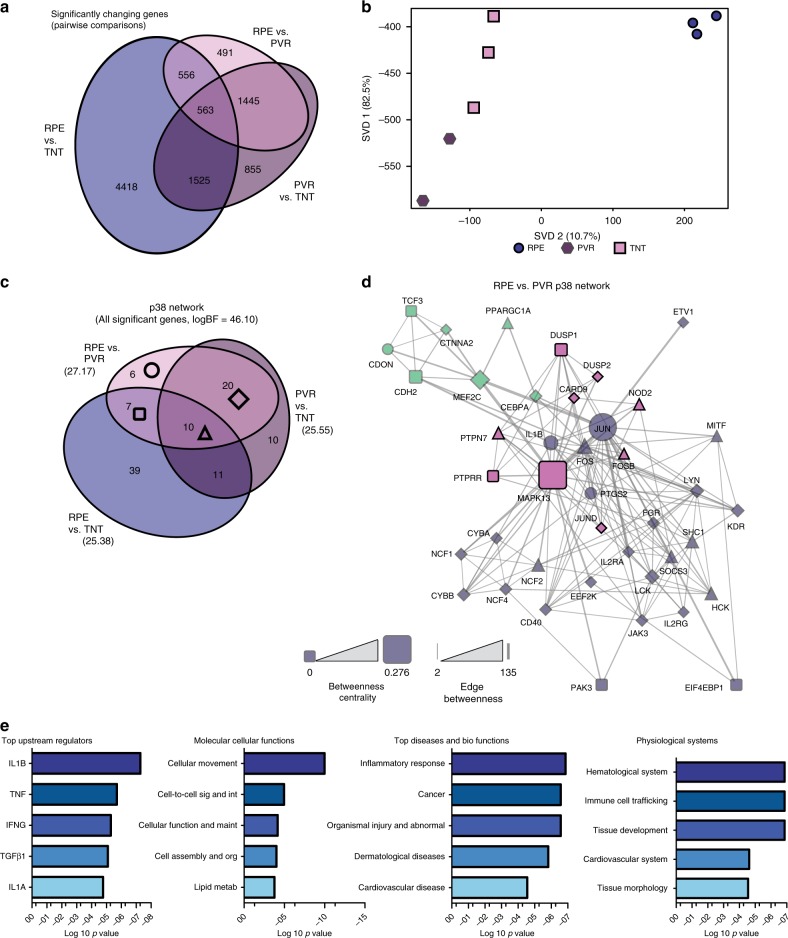
RNA expression analysis reveals a central role for a p38 network in PVR. **a** Gene expression analysis of patient-dissected PVR membranes (PVR), control ahRPE (RPE), and TNT-treated ahRPE (TNT) by RNA-sequencing. **a** Using edgeR and DEseq2 pairwise comparisons of each condition were done and overlaps between significantly changing genes were identified. **b** The data structure was examined by singular value decomposition. SVD1 and SVD2 explain ~93% of the variance in the data. **c** A p38 gene network was constructed using STRINGdb and each of the pairwise comparisons between samples was intersected with this network and an enrichment was calculated using the BayesFactor package in R (Higher BF means more evidence for enrichment). Overlaps between comparisons were identified. **d** Using the genes identified by the intersection of control ahRPE vs. patient-dissected PVR membranes comparison and the p38 network, a sub network was created. The betweenness centrality of each gene and the betweenness of each edge of this sub network was calculated and determines the size and thickness of the nodes and lines, respectively. Communities in the network were identified by the GLay algorithm (identified by color), and the shape of each node indicates the group from the Venn diagram displayed in (**c**). **e** The top categories shared between patient-dissected PVR membranes and TNT-treated ahRPE by Ingenuity pathway analysis. *Sig* signaling, *Int* interaction, *Maint* maintanence, *Org* organization, *Metab* metabolism

While expression of selected proteins and genes showed similarity between TNT-treated ahRPE and patient-dissected PVR samples, we sought to identify gene networks enriched in PVR and determine whether they are also present in TNT treated RPE. To do so, we conducted the first whole transcriptome RNA sequencing of patient-dissected PVR membranes, to our knowledge, and compared them to control and TNT-treated RPE. In TNT vs. PVR samples, there were 4388 significantly changing genes, while 2088 significantly changing genes overlapped in both comparisons (Fig. 4a), with PVR and TNT samples more similar in gene expression than RPE and TNT samples. Considering the number of significantly changing genes between the RPE vs. PVR comparison is the smallest, we suggest PVR samples may still contain a population of RPE with gene expression relatively similar to cultured RPE. To investigate this further, significantly changing genes were then analyzed by singular value decomposition (SVD)^34,35^ using the biosvd package^36^. In fact, when we graph the significantly changing genes individually in eigenspace, we find individual genes from PVR samples in close proximity to RPE (Supplementary Fig. 2). Another interesting point to note is the majority of genes from patient-dissected PVR samples are closer to genes from TNT-treated samples. The first dimension, SVD1 represents 82.5% variance of the data and the second dimension, SVD2 captures 10.7% of the variance, highlighting that SVD1 and SVD2 captures ~93% of the total gene variance (Fig. 4b).

When looking for downstream signaling convergence between TGFβ1 and TNFα by evaluating the KEGG pathway database, we identified the p38-MAPK pathway as the predominant signaling node. We therefore examined whether this pathway is enriched in our significantly changing gene comparisons. We analyzed significantly enriched signaling pathways present in both patient-dissected PVR samples and TNT-treated ahRPE. Utilizing the same gene list from the dimensional reduction described above, significantly changing genes were identified. The Bayes factor of log 46.10 highlights the confidence in the probability of the alternative hypothesis that there is a role for p38-MAPK pathway being true. Similar to the significantly changing genes above, there are 67 significantly changing involved in the p38 pathway in RPE vs. TNT and 43 significantly changing p38 implicated genes in RPE vs. PVR. Between those two comparisons, there is overlap of 7 significantly changing p38 genes (Fig. 4c). Based on the significantly changing genes identified by the intersection of control ahRPE vs. patient-dissected PVR membranes comparison and the p38 network, a sub network was constructed (Fig. 4d). The sub network highlights how significantly changing genes involved in the p38 pathway are connected. Additionally, communities can be discerned, suggesting connectedness of related genes. For instance, genes in green tend to be muscle-specific, while genes in pink are directly implicated in p38-MAPK pathway. RNA-seq analysis suggests that the p38 pathway is driving the TNT-mediated transformation of ahRPE and was enriched in patient-dissected PVR membranes. Additionally, gene ontology analysis in which significantly changing genes were upregulated in both TNT-treated ahRPE and patient-dissected PVR samples compared to control ahRPE show that both TGFβ1 and TNFα are upstream regulators of p38 and that molecular functions include cell movement and cell-to-cell signaling coupled with an inflammatory immune response (Fig. 4e). We also identified additional genes involved in muscle contraction by having at least a 2-fold increase in expression in PVR and TNT samples compared to control ahRPE by KEGG pathway analysis using the online DAVID software (Supplementary Fig. 3).

To confirm the role of p38, we evaluated candidate pathways downstream of TGFβ1 and/or TNFα by determining which signals upon TGFβ1 or TNFα alone or TNT treatment translocate to the nucleus, as they will more likely play a role in gene expression changes. p38 was the only pathway in which we observed a significant increase in nuclear localization preferentially upon TNT treatment (Supplementary Fig. 4 and Supplementary Table 2).

### Inhibition of p38 prevents TNT- induced contractile mass formation

**Fig. 5:**
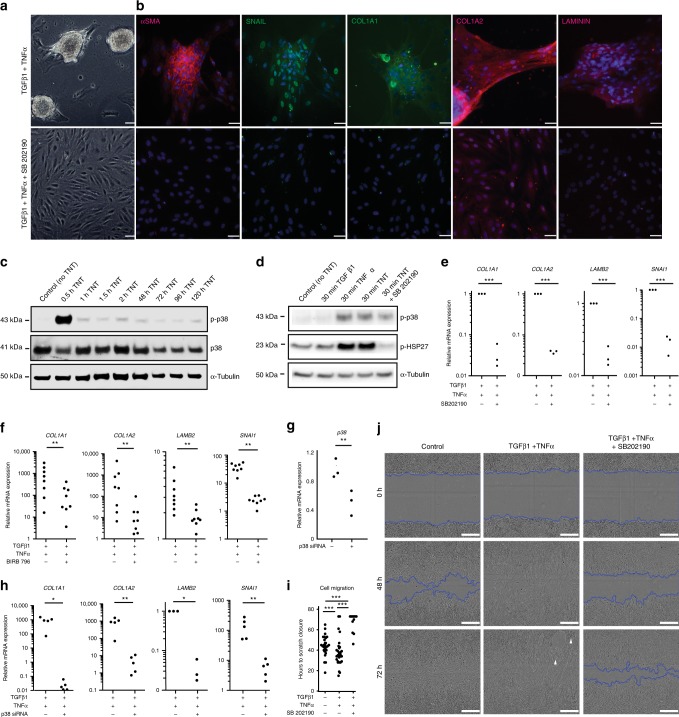
p38 inhibition in TNT-treated ahRPE. AhRPE were treated with TNT and TNT + SB 202190. **a** Phase images. Scale bar = 75 μm. **b** Anti-αSMA, SNAIL, COL1A1, COL1A2, and LAMININ immunostaining. Scale bar = 50 μm. **c** Western blot comparing phospho-p38 expression in TNT-treated ahRPE for 0 (control), 30, 60 and 120 min. **d** Western blot comparing phospho-p38 and phospho-HSP27 expression in ahRPE treated with TNT or TNT + SB 202190 for 30 min. **e** RT-qPCR of extracellular matrix and EMT genes in ahRPE treated with TNT and TNT + SB 202190. **f** RT-qPCR of extracellular matrix and EMT genes in ahRPE treated with TNT and TNT + BIRB 796. **g** RT-qPCR of p38 in cells transfected with or without p38 siRNA. **h** RT-qPCR of extracellular matrix and EMT genes in cells treated in the presence of TGFβ1 and TNFα co-supplemented basal media after transfection without p38 siRNA, and TGFβ1, TNFα co-supplemented basal media after ahRPE were transfected with p38 siRNA. Statistical significance was calculated using Wilcxon signed-rank test, *P < 0.05; **P < 0.01. **i** Quantification of wound healing scratch assay. **j** Phase images of wound healing scratch assay on ahRPE treated with basal media alone, TGFβ1 and TNFα supplemented media or TGFβ1 and TNFα and SB 202190 supplemented media for 72 h. Scale bar = 300 μm. White arrowheads indicate areas of preliminary mass formation. Statistical significance was calculated using Student’s *t*-test, *P < 0.05; **P < 0.01; and ***P < 0.001

Considering that TNT induced nuclear localization of p38-MAPK in ahRPE and that this pathway was also upregulated in patient-dissected PVR samples, we hypothesized that p38 inhibition would prevent membrane contraction. Upon TNT treatment in the presence of SB 202190, an inhibitor of the p38-MAPK pathway, ahRPE were unable to generate contractile membranes (Fig. 5a). Additionally, cells treated with TNT and SB 202190 showed decreased expression of PVR markers and EMT-associated proteins including αSMA, SNAIL, COL1A1, COL1A2, and LAMININ (Fig. 5b). Next, we hypothesized that p38 is phosphorylated and activates the p38-MAPK signaling pathway upon TNT treatment, so we evaluated p38 phosphorylation over time. Upon TNT treatment for .5, 1, 1.5, 2, 48, 72, 96, and 120 h, total protein was extracted for Western blot analysis. After 30 min, we observed a rapid and transient increase in p38 phosphorylation, which decreased by 60 min and maintained a steady state level of phosphorylation above control for up to five days (Fig. 5c). To determine whether p38 phosphorylation is essential for TNT-induced changes in ahRPE, we tested the efficacy of SB 202190, since it does not interfere with p38 phosphorylation, but instead prevents transfer of the phosphate group to downstream targets^37,38^. Heat shock protein 27 (HSP27) is a known downstream effector of p38 and has been shown to play a role in contractility^39,40^. Therefore, we tested whether inhibition of p38 by SB 202190 prevents the phosphorylation and activation of HSP27. We confirmed that upon treatment with TNT and SB 202190, HSP27 fails to be phosphorylated by p38, while p38 maintains its own phosphorylation (Fig. 5d). We evaluated mRNA expression of PVR associated genes and found treatment with TNT and SB 202190 resulted in decreased gene expression of *COL1A1* 0.034 ± 0.013, *P* ≤ 0.001, (*n* = 3), *COL1A2* 0.039 ± 0.002, *P* ≤ 0.001, (*n* = 3), *LAMB2* 0.190 ± 0.035, *P* ≤ 0.001, (*n* = 3), and *SNAI1* 0.016 ± 0.006, *P* ≤ 0.001, (mean ± S.E.M; *n* = 3) compared to TNT alone (Fig. 5e). Furthermore, BIRB 796, a p38α/β inhibitor that is structurally unrelated to SB compounds, similarly prevents TNT-induced gene expression changes. AhRPE treated with TNT and BIRB 796 showed decreased gene expression of *COL1A1* 60.7 ± 30.2, *P* ≤ 0.01, (*n* = 8), *COL1A2* 9.3 ± 4.9, *P* ≤ 0.01, (*n* = 8), *LAMB2* 1.4 ± 0.4, *P* ≤ 0.01, (*n* = 3), and *SNAI1* 1.5 ± 0.2, *P* ≤ 0.01, (mean ± S.E.M; *n* = 8) compared to TNT-treated ahRPE (Fig. 5f).

To further verify that TNT-induced mass formation is working specifically via p38 and there are no additional off-target pathways affected by p38 inhibitors^41,42^, p38 was knocked down in ahRPE using a p38 small interfering (si) RNA. p38 was knocked down approximately in half after p38 siRNA transfection for 48 h *P38* 0.59 ± 0.1, *P* = 0.0038, (mean ± S.E.M; *n* = 3) (Fig. 5g). Upon treatment with TNT and transfection with a p38 siRNA, ahRPE were unable to generate contractile membranes and showed decreased expression of *COL1A1* 10.4 ± 3.0, *P* = 0.0325, (*n* = 3), *COL1A2* 4.9 ± 1.9, *P* = 0.0062, (*n* = 5), *LAMB2* 1.7 ± 0.3, *P* = 0.0226, (*n* = 3), and *SNAI1* 6.1 ± 1.6, *P* = 0.0141, (mean ± S.E.M; *n* = 4) compared to cells treated with TNT and vehicle siRNA, further demonstrating that membrane formation and contractility are acting through a p38-MAPK signaling cascade (Fig. 5h).

In addition to contractility, another behavior hypothesized to be important in the contribution of TNT-mediated ahRPE transformation in PVR-like masses is their ability to migrate. Therefore, we tested whether p38 also plays a role in TNT-induced migration of ahRPE using a scratch-wound healing assay. TNT-treated ahRPE showed an increased rate of scratch closure 38.6 h ± 2.1, *P* ≤ 0.0001, (*n* = 43, *n* = 3 biological replicates) compared to control ahRPE 50.7 ± 2.2, *P* ≤ 0.0002, (*n* = 49, *n* = 3 biological replicates) and SB 202190 and TNT-treated ahRPE 73.1 ± 2.0, *P* ≤ 0.0001, (mean ± S.E.M; *n* = 41, *n* = 3 biological replicates) determined by the amount of time it took for cells to close the scratch wound (Figs. 5i, j).

### Inhibition of p38 reverses TNT-induced contractile mass formation

**Fig. 6:**
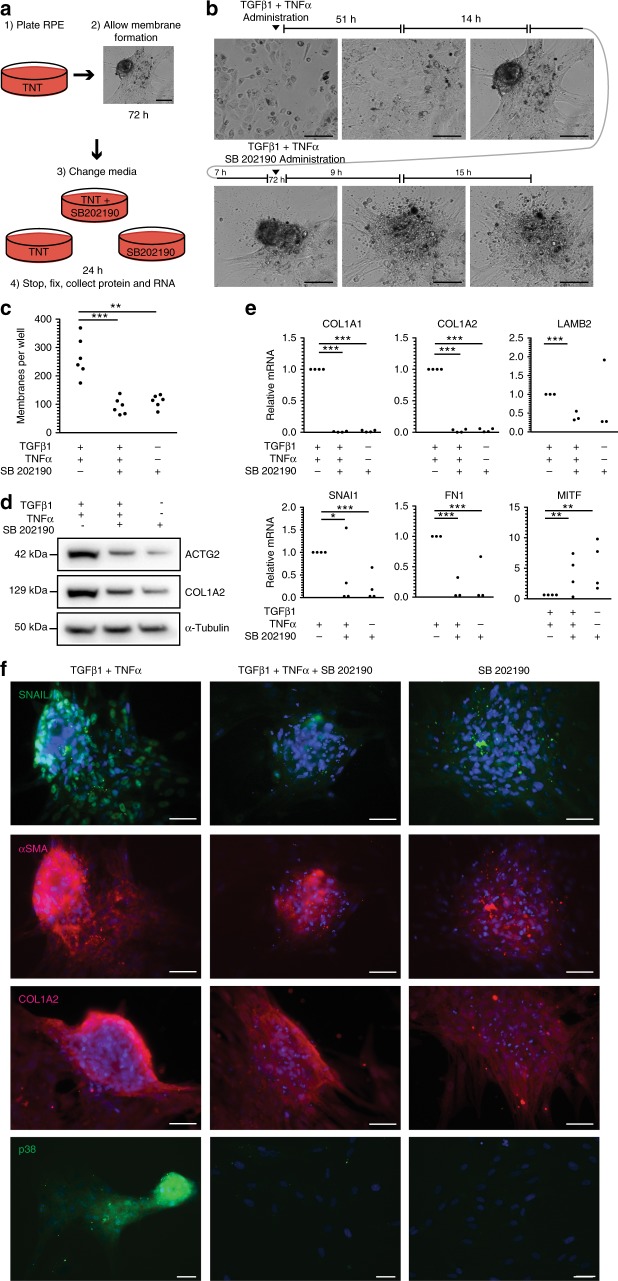
Inhibition of p38 reverses TNT-induced contractile mass formation. **a** Schematic of the experimental design to test contraction reversal. Scale bar = 100 μm. **b** Representative phase images of ahRPE treated with TNT for 72 h then media changed to TNT + SB 202190 for another 24 h. Scale bar = 100 μm. **c** Quantification of membrane formations between cultures of ahRPE in contraction reversal conditions described in 6a. **d** Western blot comparison of ACTG2 and COL1A2 between cultures of ahRPE in contraction reversal conditions. **e** RT-qPCR of extracellular matrix and EMT genes from ahRPE in contraction reversal conditions. (**f**) Anti-SNAIL, αSMA, COL1A2, and P38 immunostaining after ahRPE were placed in contraction reversal conditions. Scale bar = 75 μm. Student’s *t*-test, *P < 0.05; **P < 0.01; and ***P < 0.001

While being able to prevent PVR is advantageous and p38 inhibitors may be an effective preventative adjuvant for intravitreal surgery, patients affected by PVR often present with already formed membranes. Therefore, a principal clinical objective is to reverse already contracted membranes. We hypothesized that inhibition of p38 may relax already contracted TNT-induced ahRPE membranes. To test, we monitored contraction in TNT-treated ahRPE by time-lapse imaging in presence or not of p38 inhibitors. Following 3 days of TNT treatment, which induced mass formation, ahRPE were either treated with TNT media, TNT media supplemented with SB 202190, or basal media with SB 202190 alone. Mass contractility was then followed for an additional 24 h (Fig. 6a and Supplementary Movie 6). We found that p38 inhibition reversed contraction within 9 h (Fig. 6b). Quantification of contractile membranes showed a significant decrease in cells treated with TNT and SB 202190 81.6 ± 11.5, *P* = 0.0013, (*n* = 8) compared to TNT and a significant decrease in cells treated with SB 202190 alone 100.4 ± 9.8, *P* = 0.0034, (mean ± S.E.M; *n* = 8) compared to TNT (Fig. 6c). Additionally, Western blot analysis revealed that after robust mass formation, decreased expression of both ACTG2 and COL1A2 were found in ahRPE treated with TNT and SB 202190, or SB 202190 in basal media alone compared to cells treated with TNT, suggesting that alongside contraction reversal, there is a decrease in collagen and reversal in extracellular matrix production (Fig. 6d). This observation was validated by qPCR, which confirmed that treatment with TNT and SB 202190 resulted in decreased mRNA expression of *COL1A1* 0.008 ± .008, *P* < 0.001, (*n* = 4), *COL1A2* 0.023 ± 0.024, *P* < 0.001, (*n* = 4), *LAMB2* 0.388 ± 0.110, *P* < 0.001, (*n* = 3), *SNAI1* 0.419 ± 0.750, *P* ≤ 0.05, (*n* = 4), and *FN1* 0.039 ± 0.031, *P* < 0.001, (mean ± S.E.M; *n* = 3). Remarkably, there was increased expression of *MITF* 4.010 ± 3.107, *P* ≤ 0.01, (mean ± S.E.M; *n* = 4), a RPE specific marker, upon reversal, suggesting that the masses were returning to a RPE identity (Fig. 6e). Similarly, in membranes reversed with basal media and SB 202190 alone, there was a decrease in mRNA expression of *COL1A1* 0.020 ± .016, *P* < 0.001, (*n* = 4), *COL1A2* 0.034 ± 0.028, *P* < 0.001, (*n* = 4), *SNAI1* 0.070 ± 0.073, *P* < 0.001, (*n* = 4), and *FN1* 0.039 ± 0.010, *P* < 0.001, (mean ± S.E.M; *n* = 4). Again, there was increased expression of the RPE marker *MITF* 5.458 ± 3.923, *P* ≤ 0.01, (mean ± S.E.M; *n* = 4) (Fig. 6e). Lastly, we observed a decrease in the expression of SNAIL, αSMA, COL1A2, and P38 by immunofluorescence in cells treated with SB 202190 (Fig. 6f). In conclusion, inhibition of p38-MAPK not only prevents TNT-induced mass formation, but also reverses membranes back to a more epithelial-like, non-contractile phenotype.

### Modeling the role of RPE in PVR

**Fig. 7:**
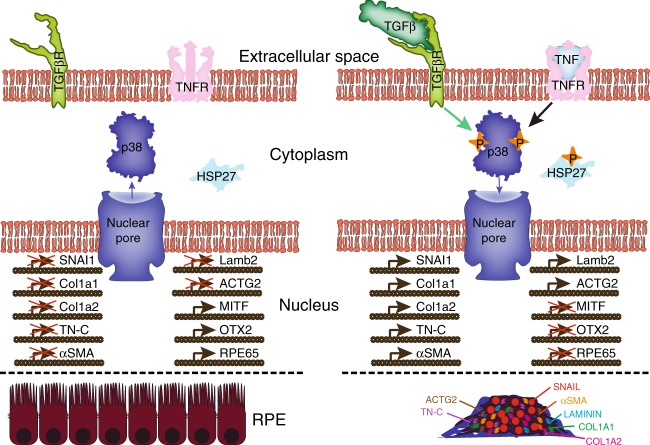
Modeling the role of RPE in PVR. When TGFβ and TNFα are not present, p38 expression is low, RPE are a polarized epithelium expressing typical RPE identity markers. Upon binding of TGFβ and TNFα by their respective receptors, p38 is phosphorylated, which leads to HSP27 phosphorylation and p38 nuclear localization. Genes associated with EMT and contractility become up-regulated and RPE identity genes are down-regulated, which leads to expression of proteins associated with PVR and RPE contraction

Based on the collection of results gathered, we propose a model of the initiation of ahRPE transformation by the synergistic action of TGFβ1 and TNFα (Fig. 7). TGFβ1 and TNFα bind to their respective receptors stimulating the phosphorylation of p38, which then indirectly phosphorylates HSP27. These responses lead to the translocation of phosphorylated p38 into the nucleus and transcriptional inhibition of RPE genes MITF, OTX2, and RPE65, and activation of an EMT program, production of membrane associated proteins and activation of contraction machinery.

## Discussion

Contractility has long been thought of as the mechanical force generator that brings PVR membranes to cause retinal detachment^43^. Identification of αSMA^44^ and Tenascin C^45^ in ERMs supported this hypothesis. We provided here additional evidence to suggest a potential contribution from RPE is in fact their ability to transdifferentiate into myocontractile-like cells. We previously discovered a putative multipotent stem cell population in RPE, which may contribute to this RPE observed cellular plasticity (Salero et al., 2012). Here we show the synergistic effect of TGFβ1 and TNFα through p38 is sufficient to stimulate RPE transformation into contractile membranes. Either TGFβ1 or TNFα alone can induce RPE to lose epithelial characteristics including the loss of E-Cadherin and the increased expression of N-Cadherin, yet only when combined do the downstream signaling of these two factors synergize to foster additional changes in ahRPE, including the expression of the mesenchymal gene SNAI1 and of muscle-associated genes, including ACTG2 and Tenascin C, as well as CDON and MEF2C, transcription factors known for muscle differentiation. Moreover, we have conducted the first whole transcriptome analysis, to our knowledge, of patient-dissected PVR samples and identified p38 MAPK central to the PVR signature network.

EMT is a multifaceted process that has been implicated in a variety of biological transformations including embryogenesis^46^, organ development^8^, fibrosis^47,48^, regeneration^49^, and cancer metastasis^50^. In this study, we find that in ahRPE, TGFβ1 and TNFα interact synergistically to drive an EMT program that mimics a step in the pathology of the blinding disease PVR. While RPE EMT is only one step in the process, the participation of RPE in ERM growth is one of the more damaging contributors, indicated by the fact that when RPE are not found in ERMs, PVR is easier to treat^3,4,51^.

Synergism between TGFβ1 and TNFα to initiate EMT has been shown in both the lung^52,53^ and intestine^54^ epithelia, but this is the first demonstration in primary ahRPE isolated from donor eyes, to our knowledge. Similarly, it has been reported that TGFβ1 and TNFα leads to the phosphorylation of p38 in colon organoids^55^ suggesting a similar signaling cascade. We demonstrate that co-stimulation by TGFβ1 and TNFα not only leads to an EMT, but also induces ahRPE cells to contract.

The role of p38 and the fibrotic response of RPE have been studied in a mouse model, in which RPE multilayering was observed in response to the removal of the /lens, vitreous, and retina^56^. Upon injection of a dominant negative form of p38, a reduction in RPE layers was observed, yet the transformation from a monolayer to multiple layers did still occur. While this model may not faithfully recapitulate PVR, the fact that DN p38 could reduce RPE response suggests p38 inhibitors may be effective in vivo at minimizing the activation of RPE observed in PVR.

In response to stress p38 translocates to the nucleus^57^. We observed nuclear localization of p38 upon TNT stimulation and during contraction. The phosphorylation of p38 occurs within 30 min of TNT stimulation, then phospho-p38 levels decline within 30 min, but stay elevated compared to control, establishing a new steady-state level. Only upon treatment with the p38 inhibitor SB 202190 p38 nuclear localization decrease, and that is concomitant with contraction reversal. Therefore, phosphorylation may transmit the switch and the nuclear localization, but maintenance of the signal may be continued by the maintenance of p38 in the nucleus, which may require TGFβ1. Based on nuclear localization experiments, the candidate pathways transmitting this additional signal may be AKT and SMAD signaling. We find both AKT and SMAD3 nuclear localization increases when both TGFβ1 and TNFα are provided compared to when either cytokine is administered alone.

Inhibiting p38 as a therapy for PVR is a promising avenue to explore. A lot of effort was placed in p38 inhibitors as treatment for many inflammatory associated diseases, including COPD^58^, rheumatoid arthritis^59^, and cancer^60^. However, many clinical trials have not progressed past Phase II due to unacceptable safety profiles. Multiple side effects have been reported, including elevated liver enzymes, skin rash, cardiotoxicity, infections, and CNS and GI toxicity^61^. However, in these cases the p38 inhibitors were provided orally and therefore the systemic, repeat exposure of p38 inhibitors likely contributed to the observed side effects. If, on the other hand, a p38 inhibitor is administered locally instead of systemically, then perhaps the severity of the side effects will be sufficiently mitigated. Although current PVR treatment does not involve the use of systemic p38 inhibition, PVR is a disease whose treatment provides the opportunity for a local, acute administration during surgery. Severe PVR is a common problem following complex retinal detachments and trauma and the only treatment is ERM removal during vitrectomy^62^. Unfortunately, PVR often returns, sometimes more aggressively. If during the initial PVR membrane removal a p38 inhibitor was administered locally, perhaps the incidence of recurrence will be reduced. Future work will explore the potential clinical approaches in utilizing local p38 inhibitors for treatment of PVR to prevent vision loss.

Clinical trials using cell replacement therapies to treat blinding diseases such as Age-Related Macular Degeneration are already underway in the United States^63^, Israel^64^, and China^65^. However, it is important to note that upon transplantation, a small incision in the retina is made to deliver the cell replacement therapy. As a result, RPE may be exposed to external cytokines, which may trigger abnormal proliferation and metaplasia as seen in ERM formation and PVR. Recently, a phase I clinical trial using hESC-derived RPE on a synthetic basement membrane resulted in one of two patients developing retinal detachment and PVR membrane formation^66^. Another clinical trial using patient specific hiPSC-derived RPE also reported pre-retinal membrane formation^67^. In each case, ERMs have never been determined to derive from the transplanted RPE. Regardless of whether ERMs are produced from the host or donor, managing p38 signaling during RPE replacement therapies may aid in improved outcomes.

To conclude, we examined the synergistic effect of TGFβ1 and TNFα on adult human RPE cultures which exhibit native physiology and compared these effects to PVR membranes dissected from patients by whole transcriptome RNA-seq. We have found RPE treated with the combination of TGFβ1 and TNFα share a gene signature with PVR membranes and central to this signature is the p38 network. Moreover, through time-lapse imaging we have identified RPE contraction upon TNT treatment. By using small molecule inhibitors and siRNA technology we were able to not only inhibit, but also reverse membrane formation and contraction. Future steps will be to test whether p38 inhibitors can prevent retinal detachment in an animal model of PVR.

## Methods

### Human adult RPE culture

Human globes from donors aged between 36 and 90 yrs were obtained from the National Disease Research Interchange, Philadelphia, PA., the Eye-Bank for Sight Restoration, Inc., New York, NY, the Lions Eye Bank, Albany, NY and Miracle in Sights, Winston-Salem, NC. Informed consent was obtained from all subjects: IRB New York Eye and Ear Infirmary of Mount Sinai Study ID#15.18. Detailed eye dissection protocols were previously published^68,69^. Globes were obtained within 40 h of death, RPE cells were isolated and plated on tissue culture plates coated with 10 μg/ml Synthemax II (Corning) in RPE medium^68^ containing Dulbecco’s Modified Eagle Medium: Nutrient Mix F-12 (DMEM/F12, Gibco), supplemented with 10% Heat Inactivated Fetal Bovine Serum (FBS, Sigma), 1X GlutaMAX (Gibco), 1X MEM Non-Essential Amino Acids Solution (Gibco), 1X Penicillin-Streptomycin (10,000 U/mL, Gibco), 1X Sodium Pyruvate (100 mM, Gibco) and 10 mM Nicotinamide (Sigma-Aldrich), which was changed 3 times a week. After the first week, FBS was reduced to 2%.

### Collection and fixation of PVR membranes

Samples included in this study derived from patients with ERMs, which were divided into a minimum of two pieces, one sent to the New York Eye and Ear Infirmary of Mount Sinai Department of Pathology and Laboratory Medicine to be PAS stained to delineate membranous components, histiocytic cytoplasmic granules, and to mask erythrocyte staining to enhance diagnostic evaluation. The other piece was sent to the Blenkinsop lab for either fixation or RNA isolation or sequencing. Samples included in this study must have, at a minimum, the presence of fibrocellular membranes of RPE origin. PVR membranes were fixed with 4% paraformaldehyde (Fisher) for 10 min, rinsed 3 times with 1X phosphate buffered saline (PBS), and placed in 30% sucrose (Sigma) for 24 h. Following fixation, membranes were embedded in Tissue-Tek® O.C.T. Compound (Sakura) and sectioned.

### Treatment of ahRPE with TNT and inhibitors

AhRPE cultures were trypsinized using 0.25% Trypsin (Gibco) for 5 min, washed and replated at 1.0 × 10^5^ cells per 200 mm^2^ per well of a 24-well plate in DMEM/F12 with 3% FBS (unless otherwise stated), 1X L-Glutamine, 1X Na-Pyruvate, 1X NEAA, 1X Pen/Strep. After 24 h, 10 ng/ml Recombinant Human Transforming Growth Factor-β1 (TGFβ1, PeproTech, Cat #:100–21) or 10 ng/ml Recombinant Human Tumor Necrosis Factor alpha (TNFα, R&D Systems, Cat #:210-TA-020) or both TGFβ1 and TNFα (TNT) were added to induce EMT and the cultures were maintained in this medium (with feeding every other day) for 5 days. SB 202190 was used at 10 ng/ml (Tocris Bioscience, Cat #:1264). BIRB 796 was used at 5.4 ng/ul (Axon Medchem, Cat #:1358). (S)-nitro-Blebbistatin was used at 100 mM (Tocris, Cat #:1852). W-7 hydrochloride was used at 50 mM (Tocris, Cat #:0369).

### Immunohistochemistry

AhRPE plated on 24 well-size transwell inserts (Corning) were fixed with 4% paraformaldehyde (Fisher) for 10 min, rinsed 3 times with phosphate buffered saline (PBS), punched using 3 mm biopsy punches (Integra) and permeabilized with 0.01% Triton X-100 (Fisher) for 20 min. Cells were then blocked in 1% BSA (Fisher) or 1% BSA supplemented with 5% Normal Goat Serum (Jackson ImmunoResearch Laboratories, Inc.) for 30 min. Primary antibody (Supplementary Table 2) in 1% BSA or 1% BSA supplemented with 5% Normal Goat Serum in PBS block was added overnight at 4 °C, then incubated with the corresponding Alexa Fluor conjugated secondary antibody (Supplementary Table 3) at room temperature for 1 h. Transwell inserts were then mounted on glass slides with Aqua-Poly/Mount (Polysciences, Inc.) and imaged were taken using a Leica microscopy. DAPI fluorescence was used to demarcate the nucleus. In order to measure nuclear fluorescence intensity, immunofluorescent images and their corresponding DAPI images were loaded into Image J. The DAPI image was used to make a mask onto the fluorescence image. The fluorescence signal intensity within each nucleus was measured and averaged for the pixels within each nucleus of each image using a scale of 0–255. Mean fluorescence measurements were then compared between control ahRPE, TGFβ1, TNFα, and TNT-treated ahRPE conditions using a paired *t*-test.

### RNA isolation and qPCR

AhRPE cells were incubated in RNAprotect Cell Reagent (Qiagen) to attenuate endogenous RNAse activity and scraped off the plate into individual 1.5 ml tubes. Cells were centrifuged at 10,000 rpms for 10 min and the pellet was re-suspended in buffer RLT plus (Qiagen). RNA was harvested from the cells according to the manufacturer protocol in the Qiagen RNeasy Plus Mini Kit. Samples were passed through a gDNA eliminator column (Qiagen) to eliminate genomic DNA. Purified total RNA was converted to cDNA using High Capacity RNA-to-cDNA Kit (Applied Biosystems) followed by qPCR with gene specific primers using using Radiant^TM^ Green 2x qPCR Mix (Alkali Scientific, Inc.) and Applied Biosystems ViiA7 Real-Time PCR System (Life Technologies). At least three independent ahRPE lines were analyzed using qPCR primers for *MITF* (Forward 5′TTGTCCATCTGCCTCTGAGTAAG; Reverse 5′CCTATGTATGACCAGGTTGCTTG), *OTX2* (Forward 5′CCATGACCTATACTCAGGCTTCAGG; Reverse 5′GAAGCTCCATATCCCTGGGTGGAAAG), *RPE65* (Forward 5′TGGTGTAGTTCTGAGTGTGGTGGT; 5′AGTCCATGAAAGGTGACAGGGATGTT), *ACTG2* (Forward 5′GCGTGTAGCACCTGAAGAG; Reverse 5′GAATGGCGACGTACATGGCA), *TENASCIN C* (Forward 5′TCCCAGTGTTCGGTGGATCT; Reverse 5′TTGATGCGATGTGTGAAGACA), *COL1A1* (Forward 5′GTGCGATGACGTGATCTGTGA; Reverse 5′CGGTGGTTTCTTGGTCGGT), *COL1A2* (Forward 5′GGCCCTCAAGGTTTCCAAGG; Reverse 5′CACCCTGTGGTCCAACAACTC), *LAMB2* (Forward 5′TGACTTTCAAGACATTCCGTCC; Reverse 5′AGGCGAAGTATCTATACACACCC), *SNAI1* (Forward 5′TGTCAGATGAGGACAGTGGGAAAGG; Reverse 5′CTGAAGTAGAGGAGAAGGACGAAGG), and *FN1* (Forward 5′AGGAAGCCGAGGTTTTAACTG; Reverse 5′AGGACGCTCATAAGTGTCACC). Expression was normalized to the endogenous control gene *PPIG* (*Cyclophilin G*, Forward 5′CTTGTCAATGGCCAACAGAGG; Reverse 5′GCCCATCTAAATGAGGAGTTGGT).

### Time-lapse microscopy

AhRPE were seeded on an uncoated 24-well plate (Corning) at a density of 100,000 cells/well in media containing DMEM/F12, 3% Heat Inactivated Fetal Bovine Serum, 1X GlutaMAX, 1X MEM Non-Essential Amino Acids Solution (Gibco), 1X Penicillin-Streptomycin, 1X Sodium Pyruvate and incubated overnight at 37 **°**C. After 24 h, cells were treated with DMEM/F12 with 3% FBS supplemented media alone or with 10 ng/ml TGFβ1, 10 ng/ml TNFα, TGFβ1 and TNFα (TNT) together or TNT supplemented with 10 ng/ml SB 201290. Following treatment, cells were imaged every 30 min for a total of 96 h in a 37 **°**C and 5% carbon dioxide humidity-controlled Leica CTR 6500 microscope chamber. Phase images were taken using Leica Microsystems LAS AF6000 software. Image analysis was performed using ImageJ software.

### Western blot

To extract protein, ahRPE were washed and then lysed with RIPA buffer containing phosphatase and protease inhibitor cocktails (Roche) on ice. Samples were scraped on ice, agitated for 30 min at 4 °C and then centrifuged at 12,000 rpm for 20 min at 4 °C to remove debris. The supernatant was collected, aliquoted to prevent freeze-thaw cycles, and protein concentration was subsequently determined using Pierce™ BCA Protein Assay Kit (Thermo Fisher Scientific). Protein extracts were separated by Bolt^TM^ 4–12% Bis-Tris Plus Gels (Thermo Fisher Scientific) and transferred onto iBlot® 2 PVDF Mini Stacks (Thermo Fisher Scientific) membranes and were probed with antibodies (Supplementary Table 4). Proteins of interest were detected with Peroxidase AffiniPure Goat Anti-Mouse IgG, F(ab’)_2_ Fragment Specific antibody (1:10000, Jackson ImmunoResearch, Cat #:115–035–072) and Peroxidase AffiniPure Goat Anti-Rabbit IgG (H + L) antibody (1:10000, Jackson ImmunoResearch, Cat #:111–035–045), and subsequently visualized with ECL^TM^ Prime Western Blotting Detection Reagent (GE Healthcare) according to the provided protocol. All samples were stored at −80 °C. Uncropped western blots for Figs. 3d, 5c, d, and 6d can be found in Supplementary Figs 5 and 6.

### Cell migration scratch assays

AhRPE cells were seeded at 16,000 cells/well in a 96-well ImageLock plate (Essen BioScience). Following 24 h, cells were scratched using a WoundMaker™ (Essen Bioscience) according to the manufacturer’s instructions, washed with basal media and treated with control media, TGFβ1- and TNFα-treated media or TNT-treated media in combination with SB 201290. Cell migration scratch assays were performed using an IncuCyte® ZOOM (software version 20162.1.6174.36615) with a Scratch Wound software module (Essen Bioscience). Experiments were conducted for 73 h with data collection every 1–2 h. Using the ×10 objective, a phase-contrast image, which fully captured the scratch wound and surrounding cellular environment was taken per well per time point. Data was analyzed using the IncuCyte software using a processing definition with the following parameters: Segmentation Adjustment—1.3; Hole fill (µm^2^)—2500; Area Min (µm^2^)—300. Wound width was calculated as an average of each vertical line of resolution of a scratch wound mask generated at a given time point.

### siRNA knockdown of p38

AhRPE cells were seeded into a 24-well plate at a density of 100,000 cells/well in media containing DMEM/F12, 3% Heat Inactivated Fetal Bovine Serum, 1X GlutaMAX, 1X MEM Non-Essential Amino Acids Solution, 1X Penicillin-Streptomycin, 1X Sodium Pyruvate and incubated overnight at 37 **°**C. Two days after initial plating, cells were transfected with 20pmol SignalSilence® p38α MAPK siRNA II (Cell Signaling Technology, Cat #:6277 S) using Lipofectamine^TM^ RNAiMAX Transfection Reagent (Thermo Fisher Scientific, Cat #: 13778–075). Following 48 h, media containing Lipofectamine Reagent and p38α MAPK siRNA II was removed. Cells were fed with basal media or media containing TGFβ1 and TNFα (TNT). Feeding was repeated every other day until visible mass formation was observed. Cells were subsequently analyzed by RT-qPCR.

### RNA sequencing

Total RNA was isolated with the Qiagen RNAeasy kit according to manufacturer instructions, and libraries were prepared using standard Illumina protocols and sequencing was performed with the Illumina HiSeq 2500 platform. After base-calling with Illumina Realtime Analysis Software, the STAR aligner was used to map read to the UCSC hg19 sparse genome (NCBI GRh37/hg19) and annotated with gencode version 19 and the counts were calculated. Counts were then imported into R and analyzed with the EdgeR and DESeq2 packages for differential gene expression. The bioSVD package was then used to provide a global picture of the significantly changing genes and generate polar plots. The p38 network was identified by taking the first and second-degree neighbors of p38 in the StringDB (Table S5). The odds of selecting a p38 network gene in our gene list was calculated by sampling over 1000 iterations. The Bayes Factor was calculated using the proportion BF function from the BayesFactor package. (Code fully available in supplement). To identify genes with altered expression between the groups, we utilized the edgeR and DEseq2 packages coupled with a 2-fold change threshold to uncover significantly changing genes. We conducted unbiased, pairwise comparisons to identify points of variation. For example, when comparing the expression of genes with a 2-fold difference between RPE and TNT-treated cells there were 7062 genes identified in either direction. SVD is a linear transformation of the expression data from the genes × arrays (samples) space to the reduced “eigengenes” × “eigenarrays” space. SVD is a useful mathematical framework for processing genome-wide expression data in which operations may be assigned a biological meaning. Therefore, SVD1 may capture cell heterogeneity as we predict this would be higher in TNT and in PVR samples while SVD2 may be capturing the EMT (Fig. 4b).

The data are diagonalized such that each eigengene is expressed only in the corresponding eigenarray with the corresponding “eigenexpression” indicating their relative significance. Using this method we can evaluate the individual genes from the different samples and map them in the “eigenarrays” space. We also took the sum of the significantly changing genes from each sample and mapped them in SVD, which is also known as Karhunen-Loeve expansion in pattern recognition^70^ and as principal-component analysis in statistics^71^. Doing so captures the variation attributed to the genes and conditions per sample as a whole and allows for an interpretation of the similarities in gene expression between the groups.

### Reporting summary

Further information on experimental design is available in the Nature Research Reporting Summary linked to this article.

## Supplementary information

Supplementary Information

Description of Additional Supplementary Files

Reporting Summary

Supplementary Movie 1

Supplementary Movie 2

Supplementary Movie 3

Supplementary Movie 4

Supplementary Movie 5

Supplementary Movie 6

## Data Availability

All raw data is available upon reasonable request. Sequencing data has been deposited on https://www.ncbi.nlm.nih.gov/geo/subs/, accession number GSE126633.
