# Supplementary material for: P38 inhibition reverses TGFβ1 and TNFα-induced contraction in a model of proliferative vitreoretinopathy
Source: Commun Biol. 2019 May 3;2:162. doi: 10.1038/s42003-019-0406-6 (PMC6499805; doi:10.1038/s42003-019-0406-6)

Supplementary Figure 1

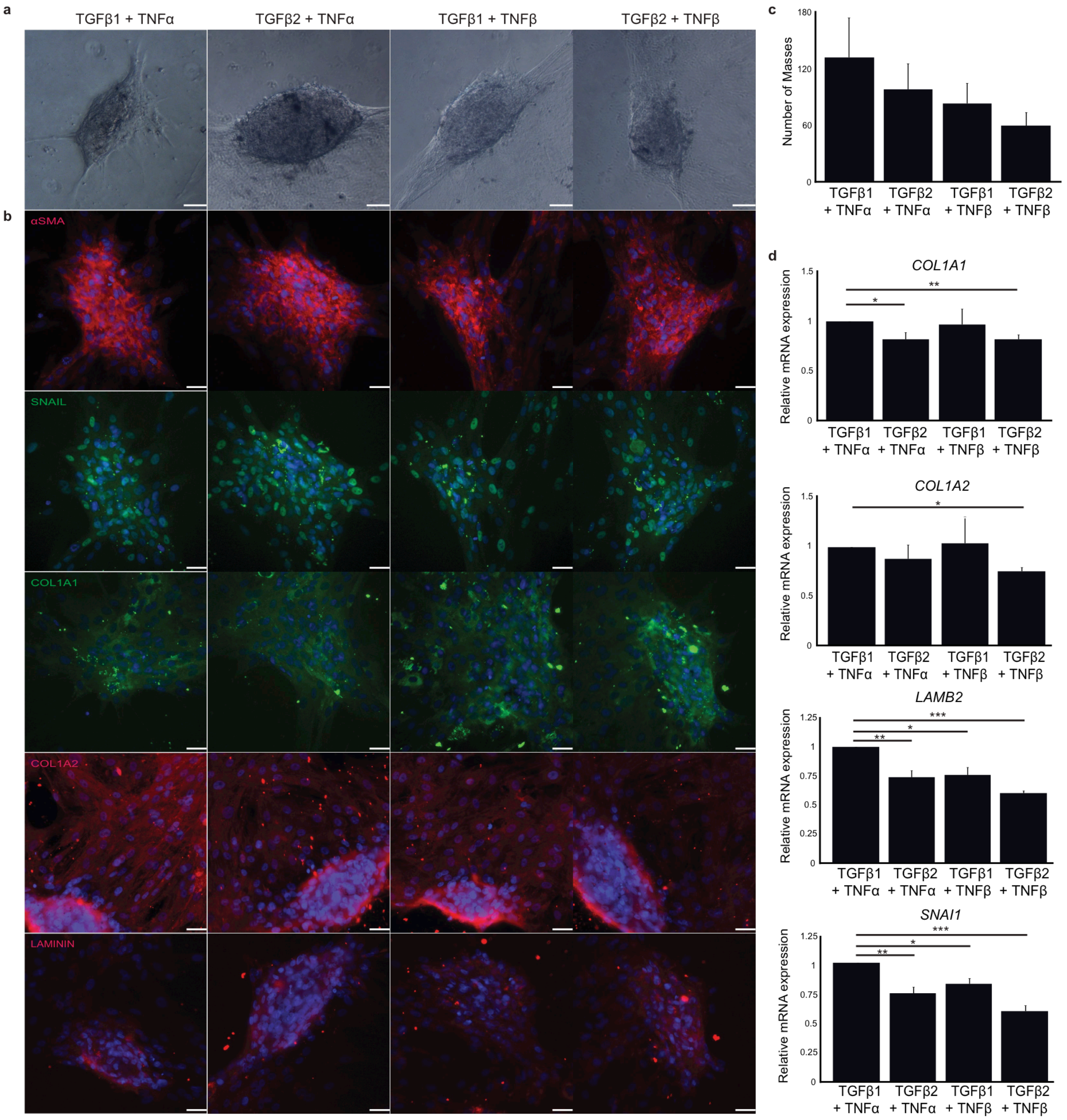

Supplementary Figure 1. Comparison of the effect of TGFβ and TNF family members on ahRPE transformation. AhRPE were cultured in the presence of 10ng/ml TGFβ1 and 10ng/ml TNFα, 10ng/ml TGFβ2 and 10ng/ml TNFα, 10ng/ml TGFβ1 and 10ng/ml TNFβ or 10ng/ml TGFβ2 and 10ng/ml TNFβ for 5 days then morphology and gene expression were analyzed. (a) Phase images. Scale bar = 50μm. (b) Anti-αSMA, SNAIL, COL1A1, COL1A2, and LAMININ immunostaining. Scale bar = 75μm. (c) Quantification of contractile membranes. (d) RT-qPCR of extracellular matrix and EMT genes. Statistical significance was calculated using Student's t-test, $^{*}P < 0.05$, $^{**}P < 0.01$ and $^{***}P < 0.001$. There was slight variation between cytokines in terms of gene expression. *COL1A1* expression decreased upon treatment with TGFβ2 + TNFα 0.814 ± 0.076, $P \leq 0.04$, ($n = 3$) and TGFβ2 + TNFβ 0.820 ± 0.047, $P \leq 0.01$, ($n = 3$), while *COL1A2* expression only decreased upon treatment with TGFβ2 + TNFβ 0.752 ± 0.105, $P \leq 0.05$, (mean +/- S.E.M; $n = 3$) compared to TNT alone. Compared to TNT treatment, *LAMB2* decreased upon all treatment conditions including TGFβ2 + TNFα 0.740 ± 0.059, $P \leq 0.05$, ($n = 3$), TGFβ1 + TNFβ 0.759 ± 0.064, $P \leq 0.05$, ($n = 3$), and TGFβ2 + TNFβ 0.601 ± 0.020, $P \leq 0.001$, (mean +/- S.E.M; $n = 3$). Similarly, *SNAI1* expression was decreased in all conditions including TGFβ2 + TNFα 0.745 ± 0.054, $P \leq 0.01$, ($n = 3$), TGFβ1 + TNFβ 0.821 ± 0.051, $P \leq 0.05$, ($n = 3$), and TGFβ2 + TNFβ 0.601 ± 0.599, $P \leq 0.001$, (mean +/- S.E.M; $n = 3$) compared to TNT treatment.

Supplementary Figure 2.

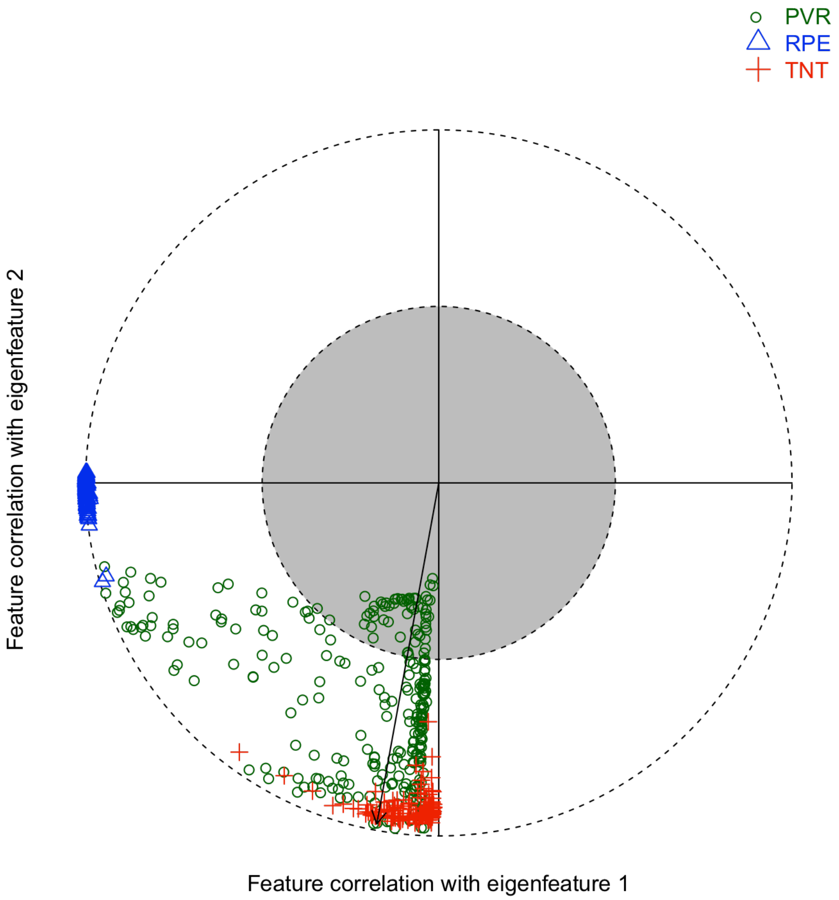

Supplementary Figure 2. Normalized elutriation expression in the subspace associated with differences with RPE changes. Feature correlation with eigenfeature2 along the y-axis vs. that with eigenfeature 1 along the x-axis, color-coded according to the sample type Blue (RPE), Red (TNT), Green (PVR). The dashed unit and half-unit circles outline 100% and 25% respectively of overall normalized array expression in the eigenfeature 1 and 2 subspace.

Supplementary Figure 3

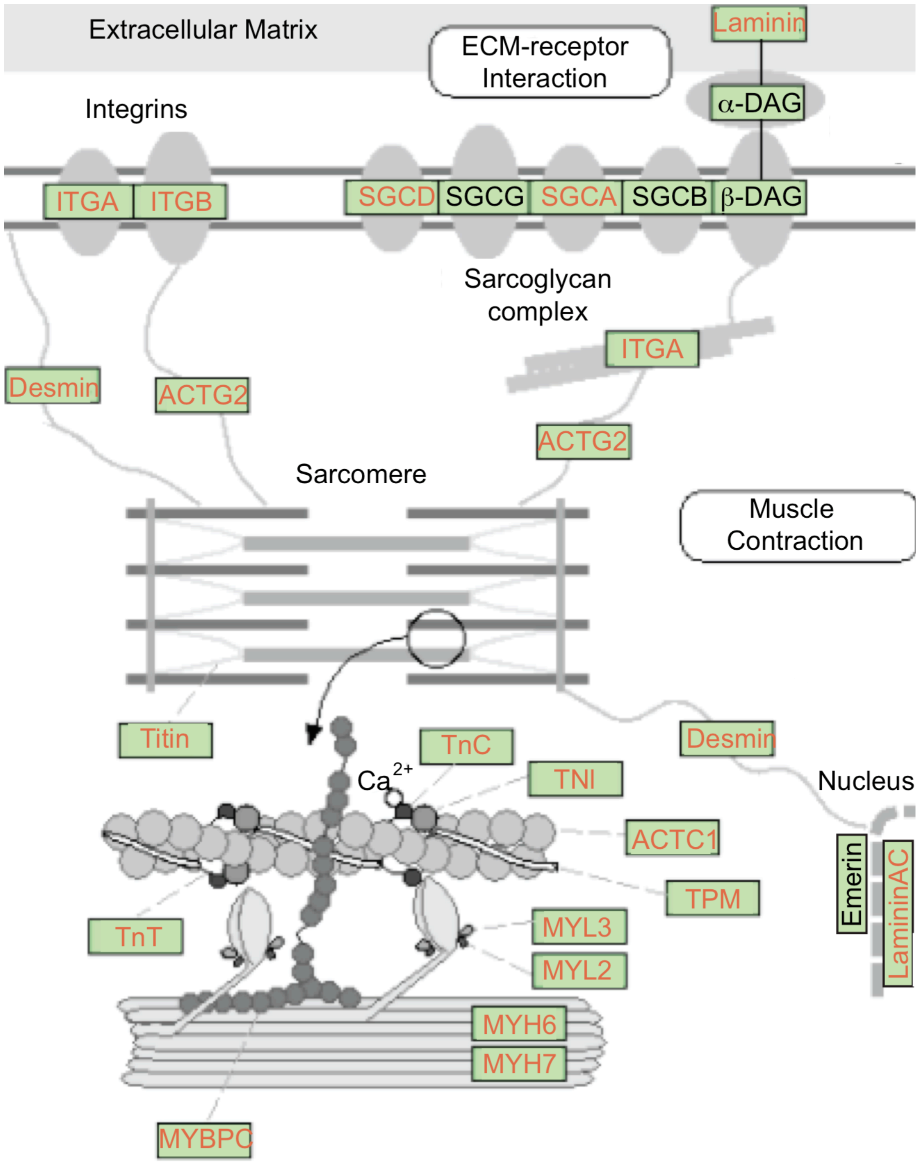

Supplementary Figure 3. KEGG pathway analysis reveals muscle gene signature. RNA-seq data from patient-dissected PVR samples and TNT-treated ahRPE were compared to control ahRPE using the KEGG pathway analysis on the online DAVID software tools (DAVID.ncifcrf.gov). KEGG pathway analysis identified genes involved in muscle contraction were enriched including ACTG2 and TENASCIN C. Genes in red indicate at least 2 fold greater expression in patient-dissected PVR samples and TNT-treated RPE compared to normal control ahRPE.

Supplementary Figure 4

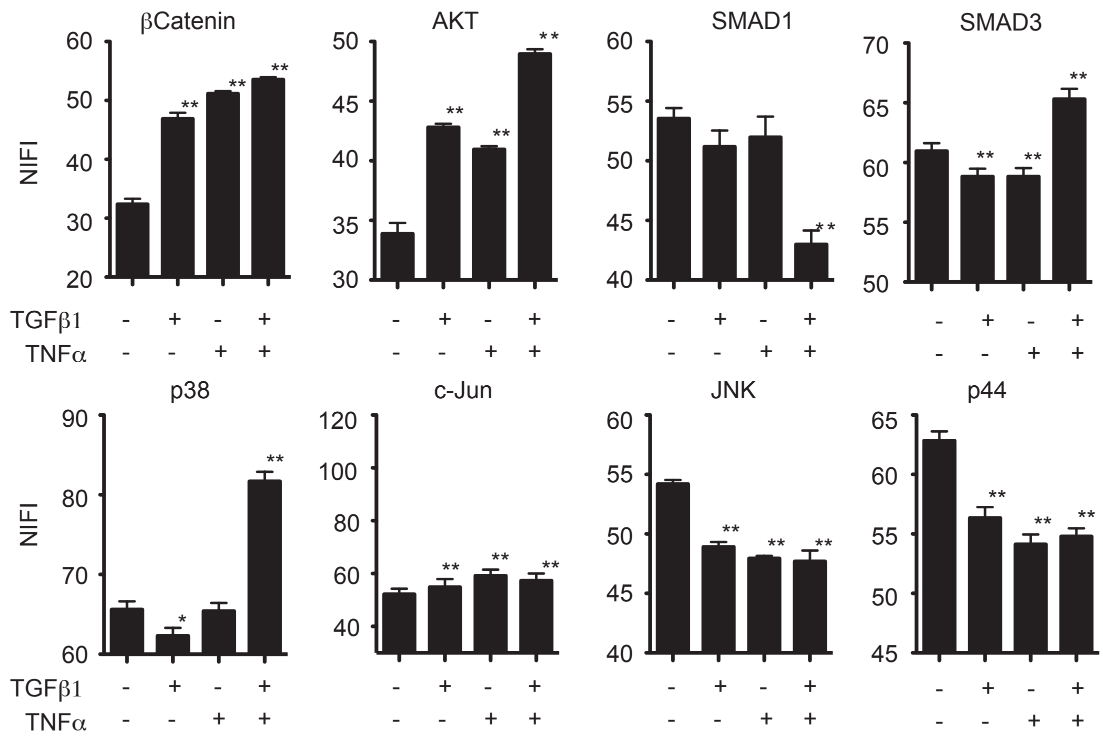

Supplementary Figure 4. p38 signaling is involved in TGFβ1 and TNFα co-activation. (a) AhRPE were cultured for 5 days in DMEM + 5% FBS in the presence of 10ng/ml TGFβ, 10ng/ml TNFα or their combination. Cells were then fixed and stained with antibodies to βCatenin, AKT, phosphorylated SMAD1, phosphorylated SMAD3, p38, c-Jun, JNK, and p44 for immunofluorescence imaging. DAPI images were used to analyze the immunofluorescence intensity of each stain exclusively within the nucleus in order to selectively measure activated forms of each protein. Nuclear immunofluorescence intensity (NIFI) was compared between conditions. Scale bar = 25 mm. (b) Anti-p38 immunostaining. Scale bar = 25μm. Student's t-test, $*P < 0.05$ and $**P < 0.01$.

Supplementary Table 1. Mass formation variability by RPE line.

RPE line	Experiment number	Mean	Coefficient of variability (%)
A	6	457.0	70.67
B	5	245.8	16.78
C	4	656.8	68.12
D	2	380.0	111.65
E	2	73.5	60.61
F	2	232.0	58.91
G	2	186.5	29.90
H	1	298.0	0
I	1	230.0	0
J	1	425.0	0

Supplementary Table 2. Nuclear immunofluorescence intensity values.

Protein	Metric	Control	TGFβ1	TNFα	TNT
**βCatenin**	mean	32.38	46.87	51.13	53.54
	SEM	0.92	1.05	0.45	0.39
	n	208	154	257	265
	P-value		<0.0001	<0.0001	<0.0001
**AKT**	mean	33.87	42.81	40.96	48.95
	SEM	0.92	0.29	0.25	0.402
	n	288	169	258	273
	P-value		<0.001	<.001	<.001
**SMAD1**	mean	53.53	51.18	51.97	42.99
	SEM	0.92	1.05	0.45	0.39
	n	208	154	257	265
	P-value		0.15	0.47	<0.0001
**SMAD3**	mean	61.85	58.82	58.82	65.3
	SEM	0.67	0.67	0.71	0.87
	n	186	172	224	359
	P-value		0.0016	0.0024	0.0085
**p38**	mean	65.61	62.31	65.4	81.69
	SEM	1.02	0.99	1.03	1.17
	n	144	161	125	167
	P-value		0.0216	0.88	<0.0001
**cJun**	mean	54.02	56.73	59.3	56.53
	SEM	0.26	0.66	0.512	0.64
	n	589	237	468	431
	P-value		<0.0001	<0.0001	<0.0001
**JNK**	mean	54.19	48.9	47.94	47.69
	SEM	0.34	0.42	0.21	0.9
	n	398	351	419	416
	P-value		<0.0001	<0.0001	<0.0001
**p44**	mean	62.83	56.34	54.12	54.79
	SEM	0.78	0.9	0.83	0.67
	n	274	231	380	356
	P-value		<0.0001	<0.0001	<0.0001

* P-value relative to control conditions.

Supplementary Table 3. Antibodies and working dilutions.

Antibodies use for immunofluorescence

Target	Host Species	Source	Catalog Number	Working Dilution
ACTG2	Rabbit	Abcam	ab209694	1:100
TENASCIN C	Rabbit	Abcam	ab108930	1:100
αSMA	Mouse	Abcam	ab7817	1:100
SNAIL	Goat	R&D Systems	AF3639	1:200
COL1A1/PICP	Mouse	Abcam	ab76102	1:50
COL1A2	Rabbit	Abcam	ab96723	1:50
LAMININ	Rat	Abcam	ab44941	1:20
AKT	Rabbit	Cell Signaling	9271	1:100
b-Catenin	Mouse	BD Biosciences	610154	1:100
cJun	Rabbit	Cell Signaling	91653	1:100
JNK	Mouse	Cell Signaling	9255	1:100
p38	Rabbit	Cell Signaling	9211S	1:100
p44	Rabbit	Millipore	05-797R	1:100
pSMAD3	Rabbit	Cell Signaling	9523	1:100
SMAD1	Rabbit	Cell Signaling	9743	1:100

Supplementary Table 4. Antibodies and working dilutions.

Secondary antibodies used for immunofluorescence

Target	Host Species	Source	Target	Host Species
Alexa Fluor® 546 Goat anti-Rabbit IgG (H+L)	Rabbit	Thermo Fisher	A-11071	1:1000
Alexa Fluor® 546 Goat anti-Mouse IgG (H+L)	Mouse	Thermo Fisher	A-11018	1:1000
Alexa Fluor® 488 Donkey anti-Goat IgG (H+L)	Goat	Thermo Fisher	A-11055	1:1000
Alexa Fluor® 488 Goat anti-Mouse IgG (H+L)	Mouse	Thermo Fisher	A-11017	1:1000
Alexa Fluor® 647 Goat anti-Rat IgG (H+L)	Rat	Abcam	Ab150159	1:1000
DAPI (4',6-Diamidine-2'-phenylindole dihydrochloride)	Rabbit	Thermo Fisher	10 236 276 001	1:1000

Supplementary Table 5. Antibodies and working dilutions

Antibodies used for Western blot

Target	Host Species	Source	Catalog Number	Working Dilution
E-Cadherin	Rabbit	Cell Signaling	3195	1:1000
N-Cadherin	Rabbit	Abcam	ab12221	1:100
Vinculin	Rabbit	Abcam	ab129002	1:10000
phospho-p38	Rabbit	Cell Signaling	9211S	1:1000
phospho-HSP27	Rabbit	Abcam	ab155987	1:1000
α-Tubulin	Mouse	Sigma-Aldrich	T9026	1:5000
ACTG2	Rat	Abcam	ab209694	1:500
COL1A2	Rabbit	Abcam	ab96723	1:500

Supplementary Blot 1

Figure 3d

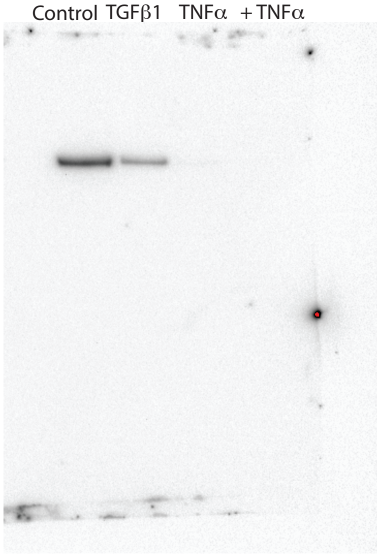

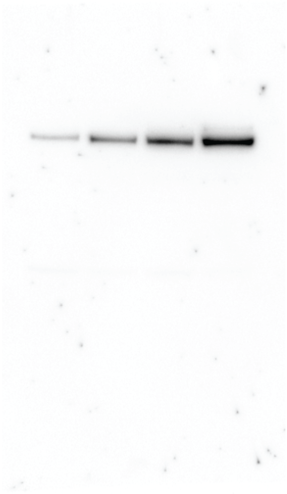

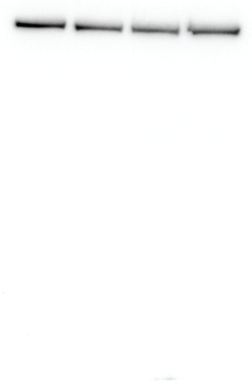

Supplementary Blot 2

Figure 5c

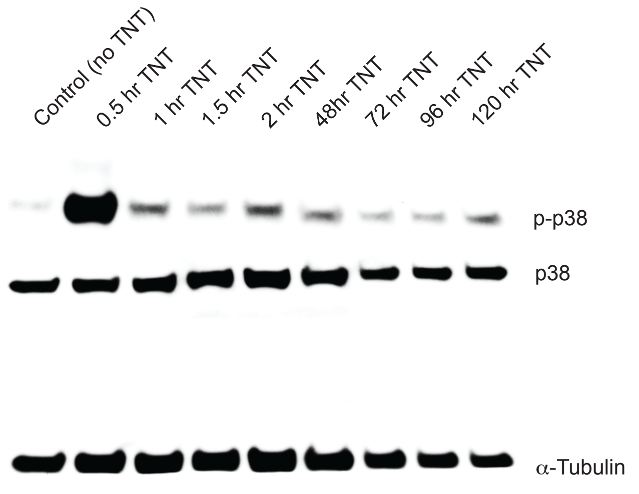

Single blot cut into 3 pieces

Supplementary Blot 3

Figure 5d

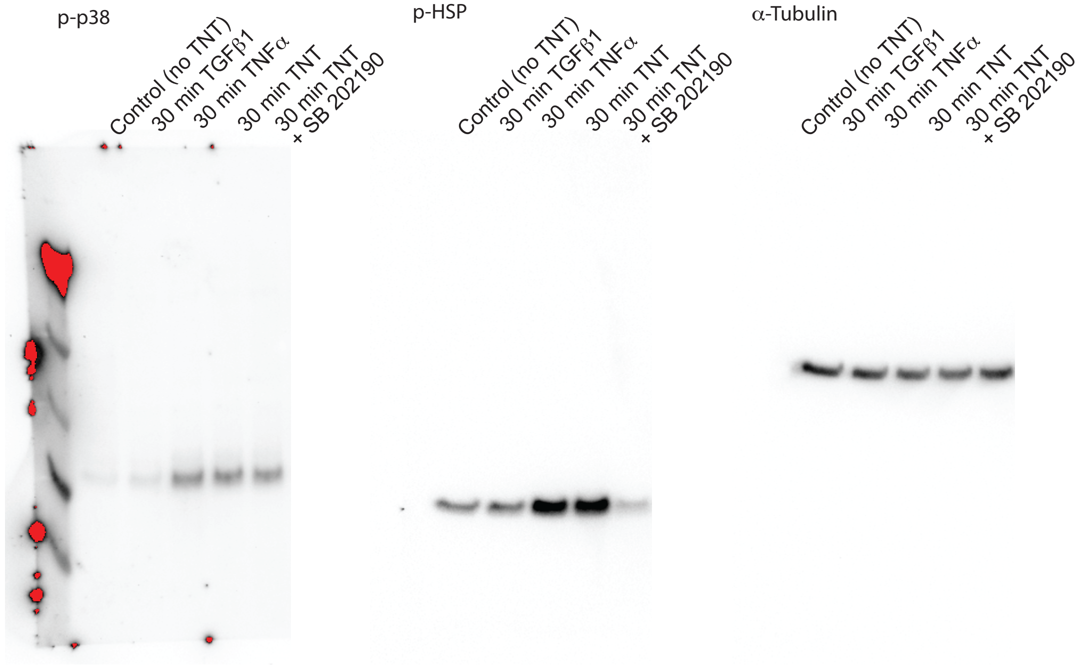

Supplementary Blot 4

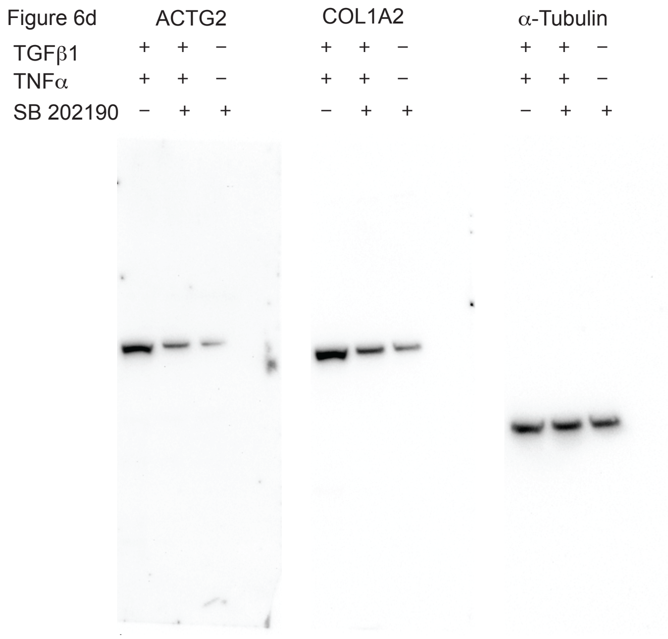

Supplement: Supplementary file 1 — Supplementary Information [file 42003_2019_406_MOESM1_ESM.pdf]
