# Supplementary material for: P38 inhibition reverses TGFβ1 and TNFα-induced contraction in a model of proliferative vitreoretinopathy
Source: Commun Biol. 2019 May 3;2:162. doi: 10.1038/s42003-019-0406-6 (PMC6499805; doi:10.1038/s42003-019-0406-6)
Supplement: Supplementary file 2 — Description of Additional Supplementary Files [file 42003_2019_406_MOESM2_ESM.pdf]

Description of Additional Supplementary Files

File Name: Supplementary Movie 1

Description: **Time-lapse of ahRPE treated with FBS-basal control media**. AhRPE were seeded on an uncoated 24-well plate at a density of 100,000 cells/well in media containing DMEM/F12 and 3% Heat Inactivated Fetal Bovine Serum (FBS) and then incubated overnight at 37°C. After 24 hours, cells were treated with DMEM/F12 with 3% FBS media. Following treatment, cells were imaged every 30 minutes for a total of 96 hours in a 37°C and 5% carbon dioxide humidity-controlled Leica CTR 6500 microscope chamber. Phase images were taken using Leica Microsystems LAS AF6000 software. Image analysis was performed using ImageJ software. Scale bar = 300µm.

File Name: Supplementary Movie 2

Description: **Time-lapse of ahRPE treated with FBS-basal media supplemented with TGFβ1.** AhRPE were seeded on an uncoated 24-well plate at a density of 100,000 cells/well in media containing DMEM/F12 and 3% Heat Inactivated Fetal Bovine Serum (FBS) and then incubated overnight at 37°C. After 24 hours, cells were treated with DMEM/F12 with 3% FBS media supplemented with 10 ng/ml TGFβ1. Following treatment, cells were imaged every 30 minutes for a total of 96 hours in a 37°C and 5% carbon dioxide humidity-controlled Leica CTR 6500 microscope chamber. Phase images were taken using Leica Microsystems LAS AF6000 software. Image analysis was performed using ImageJ software. Scale bar = 300µm.

File Name: Supplementary Movie 3

Description: **Time-lapse of ahRPE treated with FBS-basal media supplemented with TNFα.** AhRPE were seeded on an uncoated 24-well plate at a density of 100,000 cells/well in media containing DMEM/F12 and 3% Heat Inactivated Fetal Bovine Serum (FBS) and then incubated overnight at 37°C. After 24 hours, cells were treated with DMEM/F12 with 3% FBS media supplemented with 10 ng/ml TNFα. Following treatment, cells were imaged every 30 minutes for a total of 96 hours in a 37°C and 5% carbon dioxide humidity-controlled Leica CTR 6500 microscope chamber. Phase images were taken using Leica Microsystems LAS AF6000 software. Image analysis was performed using ImageJ software. Scale bar = 300µm.

File Name: Supplementary Movie 4

Description: **Time-lapse of ahRPE treated with FBS-basal media co-supplemented with TGFβ1 and TNFα.** AhRPE were seeded on an uncoated 24-well plate at a density of 100,000 cells/well in media containing DMEM/F12 and 3% Heat Inactivated Fetal Bovine Serum (FBS) and then incubated overnight at 37°C. After 24 hours, cells were treated with DMEM/F12 with 3% FBS media co-supplemented with 10 ng/ml TGFβ1 and 10 ng/ml TNFα. Following treatment, cells were imaged every 30 minutes for a total of 96 hours in a 37°C and 5% carbon dioxide humidity-controlled Leica CTR 6500 microscope chamber. Phase images were taken using Leica Microsystems LAS AF6000 software. Image analysis was performed using ImageJ software. Scale bar = 300µm.

File Name: Supplementary Movie 5

Description: **Time-lapse of ahRPE treated with FBS-basal media cosupplemented with TGFβ1 and TNFα.** AhRPE were seeded on an uncoated 24-well plate at a density of 100,000 cells/well in media containing DMEM/F12 and 3% Heat Inactivated Fetal Bovine Serum (FBS) and then incubated overnight at 37°C. After 24 hours, cells were treated with DMEM/F12 with 3% FBS media co-supplemented with 10 ng/ml TGFβ1 and 10 ng/ml TNFα. Following treatment, cells were imaged every 30 minutes for a total of 96 hours in a 37°C and 5% carbon dioxide humidity-controlled Leica CTR 6500 microscope chamber. Phase images were taken using Leica Microsystems LAS AF6000 software. Image analysis was performed using ImageJ software. Scale bar = 300μm.

File Name: Supplementary Movie 6

Description: **Time-lapse of 3D mass reversal using p38 inhibitor, SB 202190.** AhRPE were seeded on an uncoated 24-well plate at a density of 100,000 cells/well in media containing DMEM/F12 and 3% Heat Inactivated Fetal Bovine Serum (FBS) and then incubated overnight at 37°C. After 24 hours, cells were treated with DMEM/F12 with 3% FBS media co-supplemented with 10 ng/ml TGFβ1 and 10 ng/ml TNFα and imaged every 30 minutes for 96 hours in a 37°C and 5% carbon dioxide humidity-controlled Leica CTR 6500 microscope chamber. Following robust mass formation after 3 days, cells were treated with 10 ng/ml TGFβ1, 10 ng/ml TNFα, and 10 ng/ml SB 201290 and further imaged every 30 minutes for an additional 96 hours. Phase images were taken using Leica Microsystems LAS AF6000 software. Image analysis was performed using ImageJ software. Scale bar = 300μm.
