# Supplementary material for: P38 inhibition reverses TGFβ1 and TNFα-induced contraction in a model of proliferative vitreoretinopathy
Source: Commun Biol. 2019 May 3;2:162. doi: 10.1038/s42003-019-0406-6 (PMC6499805; doi:10.1038/s42003-019-0406-6)
Supplement: Supplementary file 3 — Reporting Summary [file 42003_2019_406_MOESM3_ESM.pdf]

nature research

Corresponding author(s): Timothy A. Blenkinsop

Reporting Summary

Nature Research wishes to improve the reproducibility of the work that we publish. This form provides structure for consistency and transparency in reporting. For further information on Nature Research policies, see Authors & Referees and the Editorial Policy Checklist.

Statistical parameters

When statistical analyses are reported, confirm that the following items are present in the relevant location (e.g. figure legend, table legend, main text, or Methods section).

n/a	Confirmed	
☐	☒	The exact sample size (*n*) for each experimental group/condition, given as a discrete number and unit of measurement
☐	☒	An indication of whether measurements were taken from distinct samples or whether the same sample was measured repeatedly
☐	☒	The statistical test(s) used AND whether they are one- or two-sided<br>*Only common tests should be described solely by name; describe more complex techniques in the Methods section.*
☐	☒	A description of all covariates tested
☐	☒	A description of any assumptions or corrections, such as tests of normality and adjustment for multiple comparisons
☒	☐	A full description of the statistics including central tendency (e.g. means) or other basic estimates (e.g. regression coefficient) AND variation (e.g. standard deviation) or associated estimates of uncertainty (e.g. confidence intervals)
☐	☒	For null hypothesis testing, the test statistic (e.g. *F*, *t*, *r*) with confidence intervals, effect sizes, degrees of freedom and *P* value noted<br>*Give P values as exact values whenever suitable.*
☒	☐	For Bayesian analysis, information on the choice of priors and Markov chain Monte Carlo settings
☒	☐	For hierarchical and complex designs, identification of the appropriate level for tests and full reporting of outcomes
☒	☐	Estimates of effect sizes (e.g. Cohen's *d*, Pearson's *r*), indicating how they were calculated
☐	☒	Clearly defined error bars<br>*State explicitly what error bars represent (e.g. SD, SE, CI)*

Our web collection on statistics for biologists may be useful.

Software and code

Policy information about availability of computer code

Data collection	Realtime Analysis Software, STAR aligner, EdgeR, DESeq2, BioSVD package, StringDB, BayesFactor package
Data analysis	STAR aligner, EdgeR, DESeq2, BioSVD package, StringDB, BayesFactor package, Prism Graphpad

For manuscripts utilizing custom algorithms or software that are central to the research but not yet described in published literature, software must be made available to editors/reviewers upon request. We strongly encourage code deposition in a community repository (e.g. GitHub). See the Nature Research guidelines for submitting code & software for further information.

Data

Policy information about availability of data

All manuscripts must include a data availability statement. This statement should provide the following information, where applicable:

- Accession codes, unique identifiers, or web links for publicly available datasets
- A list of figures that have associated raw data
- A description of any restrictions on data availability

Sequence data that support the findings of this study have been deposited in GEO Datasets with the primary accession code GSE126633 https://www.ncbi.nlm.nih.gov/geo/subs/

Field-specific reporting

Please select the best fit for your research. If you are not sure, read the appropriate sections before making your selection.

☒ Life sciences ☐ Behavioural & social sciences ☐ Ecological, evolutionary & environmental sciences

For a reference copy of the document with all sections, see nature.com/authors/policies/ReportingSummary-flat.pdf

Life sciences study design

All studies must disclose on these points even when the disclosure is negative.

Sample size	Minimum 3 genetically distinct biological samples were used for all analyses.
Data exclusions	No data was excluded
Replication	All experiments were conducted a minimum on three biologicall replicates.
Randomization	Samples were collected by the random opportunity of receiving donor tissue through eye banks.
Blinding	Samples were encoded with barcodes before analyses, then decoded after data was analyzed.

Reporting for specific materials, systems and methods

Materials & experimental systems

n/a	Involved in the study	
☐	☒	Unique biological materials
☐	☒	Antibodies
☐	☒	Eukaryotic cell lines
☒	☐	Palaeontology
☒	☐	Animals and other organisms
☒	☐	Human research participants

Methods

n/a	Involved in the study	
☒	☐	ChIP-seq
☒	☐	Flow cytometry
☒	☐	MRI-based neuroimaging

Unique biological materials

Policy information about availability of materials

Obtaining unique materials	human globes were received after consent by either the donor or the next of kin from the National Disease Research Interchange, Philadelphia, PA., the Eye-Bank for Sight Restoration, Inc., New York, NY, the Lions Eye Bank, Albany, NY and Miracle in Sights, Winston-Salem, NC. Informed consent was obtained from all subjects.

Antibodies

Antibodies used

ACTG2 Rabbit Abcam ab209694 1:100
TENASCIN C Rabbit Abcam ab108930 1:100
αSMA Mouse Abcam ab7817 1:100
SNAIL Goat R&D Systems AF3639 1:200
COL1A1/PICP Mouse Abcam ab76102 1:50
COL1A2 Rabbit Abcam ab96723 1:50
LAMININ Rat Abcam ab44941 1:20
AKT Rabbit Cell Signaling 9271 1:100
[illegible]-Catenin Mouse BD Biosciences 610154 1:100
cJun Rabbit Cell Signaling 91653 1:100
JNK Mouse Cell Signaling 9255 1:100
p38 Rabbit Cell Signaling 9211S 1:100
p44 Rabbit Millipore 05-797R 1:100
pSMAD3 Rabbit Cell Signaling 9523 1:100
SMAD1 Rabbit Cell Signaling 9743 1:100

	Target Host Species Source Target Host Species<br>Alexa Fluor® 546 Goat anti-Rabbit IgG (H+L) Rabbit Thermo Fisher A-11071 1:1000<br>Alexa Fluor® 546 Goat anti-Mouse IgG (H+L) Mouse Thermo Fisher A-11018 1:1000<br>Alexa Fluor® 488 Donkey anti-Goat IgG (H+L) Goat Thermo Fisher A-11055 1:1000<br>Alexa Fluor® 488 Goat anti-Mouse IgG (H+L) Mouse Thermo Fisher A-11017 1:1000<br>Alexa Fluor® 647 Goat anti-Rat IgG (H+L) Rat Abcam Ab150159 1:1000<br>DAPI (4’,6-Diamidine-2’-phenylindole dihydrochloride) Rabbit Thermo Fisher 10 236 276 001 1:1000
Validation	Antibodies used were purchased from companies that validate. we validate annually are the antibodies used. We have validated the majority of antibodies proposed in this application in the following way and those yet to be validated will be authenticated similarly. Antibodies are only purchased from select vendors, which display examples of the staining. Once purchased, the same conditions are replicated in lab, which includes concentration, vehicle and tissue or cells used as a positive control. If antibodies are directed against proteins known to be generally present in all cells, then no negative control is used, but cellular localization is used to demonstrate consistent staining pattern. If an antibody is cell-specific, then cells known to not express that target protein are used as a negative control, alongside experimental conditions. In all experiments specific isotype controls are used for each antibody as well as staining with secondary antibody alone.

Eukaryotic cell lines

Policy information about cell lines

Cell line source(s)	We derive each RPE line from an individual cadaver donor globes, 26 lines of which were used in the experiments presented in this manuscript.
Authentication	Each cell line is validated by by testing purity, identity and potency using immunofluorescence, qPCR and electrophysiological assays.
Mycoplasma contamination	Mycoplasma is routinely tested on all lines maintained.
Commonly misidentified lines<br>(See ICLAC register)	N/A
